# Recombination-mediated remodelling of host–pathogen interactions during *Staphylococcus aureus* niche adaptation

**DOI:** 10.1099/mgen.0.000036

**Published:** 2015-10-30

**Authors:** Laura E. Spoor, Emily Richardson, Amy C. Richards, Gillian J. Wilson, Chriselle Mendonca, Ravi Kr. Gupta, Paul R. McAdam, Stephen Nutbeam-Tuffs, Nikki S. Black, James P. O'Gara, Chia Y. Lee, Jukka Corander, J. Ross Fitzgerald

**Affiliations:** ^1^​ The Roslin Institute and Edinburgh Infectious Diseases, University of Edinburgh, Easter Bush, Midlothian, UK; ^2^​ Department of Microbiology and Immunology, University of Arkansas School for Medical Sciences, Little Rock, Arkansas, USA; ^3^​ Department of Microbiology, School of Natural Sciences, National University of Ireland Galway, Ireland; ^4^​ Department of Mathematics and Statistics, University of Helsinki, Helsinki, Finland

**Keywords:** host–pathogen interactions, niche adaptation, recombination, remodelling, *Staphylococcus aureus*

## Abstract

Large-scale recombination events have led to the emergence of epidemic clones of several major bacterial pathogens. However, the functional impact of the recombination on clonal success is not understood. Here, we identified a novel widespread hybrid clone (ST71) of livestock-associated *Staphylococcus aureus* that evolved from an ancestor belonging to the major bovine lineage CC97, through multiple large-scale recombination events with other *S. aureus* lineages occupying the same ruminant niche. The recombination events, affecting a 329 kb region of the chromosome spanning the origin of replication, resulted in allele replacement and loss or gain of an array of genes influencing host–pathogen interactions. Of note, molecular functional analyses revealed that the ST71 hybrid clone has acquired multiple novel pathogenic traits associated with acquired and innate immune evasion and bovine extracellular matrix adherence. These findings provide a paradigm for the impact of large-scale recombination events on the rapid evolution of bacterial pathogens within defined ecological niches.

## Data Summary

1. Genome sequences from the current study have been deposited in the European Nucleotide Archive under accession numbers ERS515451–ERS515452 with the study accession number PRJEB6888 (http://www.ebi.ac.uk/ena/data/view/PRJEB6888).

## Impact Statement

Large-scale recombination events involving the transfer of large fragments of DNA between strains of bacteria have led to the emergence of new epidemic hybrid clones. In some cases, such events have been linked to capsule serotype switching, but the broader functional impact and role in epidemic success is not well understood. Here, we identified a novel hybrid clone of livestock-associated *Staphylococcus aureus* that has evolved by replacement of a large region of the genome via multiple genetic imports from other *S. aureus* strains in the same niche. Functional analysis reveals that the recombination has led to the gain of several pathogenic traits associated with immune evasion and host–pathogen interactions. The study demonstrates for the first time how large-scale recombination events can have a profound impact on the phenotype and fitness of bacteria within defined environmental niches. The findings have broad implications for the role of such events in the emergence and success of other hybrid clones of pathogenic bacteria.

## Introduction

Recently, whole-genome sequencing studies have revealed large-scale (macro-) recombination events linked to the emergence of epidemic clones of an array of major bacterial pathogens (Brochet *et al.*, 2008; Chen *et al.*, 2014; de Been *et al.*, 2013; Mostowy *et al.*, 2014; Robinson & Enright, 2004). For example, the epidemic clone defined by sequence type ST258 of *Klebsiella pneumoniae* responsible for the recent global spread of carbapenem-resistant *K. pneumoniae* is a hybrid clone resulting from a recombination event of a 1.1 Mbp chromosomal fragment from an ST442-like strain with 4.2 Mbp derived from an ST11-like strain (Chen *et al.*, 2014). In addition, large recombination events involving acquisition by conjugation of chromosomal segments up to 300 kb regions have driven the emergence of major human clinical clones of *Streptococcus agalactiae* (Brochet *et al.*, 2008). Furthermore, large recombination events have been linked to capsule serotype switching and antibiotic resistance among *Streptococcus pneumoniae* strains in response to vaccination, and *Enterococcus faecium* clones undergoing hospital adaptation (de Been *et al.*, 2013; Mostowy *et al.*, 2014). Although *Staphylococcus aureus* is a highly clonal organism, a recent species-wide comparative genomic study identified that chromosomal regions flanking mobile genetic elements and a ∼750 kb region spanning the origin of replication (*oriC*) had an elevated recombination rate (Everitt *et al.*, 2014). This is consistent with the previous discovery that the pandemic human hospital-associated meticillin-resistant *S. aureus* CC239 clone evolved from an import of a 635 kb region from a CC30 donor strain into a CC8 genetic background in a region spanning the origin (Robinson & Enright, 2004; Smyth *et al.*, 2010). Similar, large-scale homologous recombination events of ∼250 kb in size have resulted in hybrid genomes for human *S. aureus* clones ST34 and ST42 (Robinson & Enright, 2004). Although the emergence and expansion of hybrid clones has been linked to the recombinant events that define them, our understanding of the influence of such events on the ecological success of bacterial pathogens is not well understood. Here, we report the identification of a widespread livestock-associated hybrid clone of *S. aureus* which emerged from the major bovine clone of *S. aureus* CC97 via multiple, large recombination events with other *S. aureus* strains occupying the ruminant niche. Furthermore, we investigate the consequences of the recombination events on host–pathogen interactions, identifying multiple gains of function that contribute to bovine immune evasion and enhanced host–pathogen interactions. Our data highlight the potential impact of large-scale recombination events on the emergence of new bacterial clones within defined environmental niches.

## Methods

### Bacterial culture and genomic DNA isolation

All bacterial strains employed are listed in Table S1 (available in the online Supplementary Material). The ST71 *S. aureus* strains sequenced in the current study were isolated from cases of bovine mastitis in Ireland in 1993 (RF103) and the UK in 2003 (C01122). Strain Phillips and the Cna (collagen adhesin)-deficient mutant were constructed and described previously (Patti *et al.*, 1994). For phylogenetic analysis, the ST71 *S. aureus* strains were added to a previously defined dataset of CC97 *S. aureus* (Spoor *et al.*, 2013). For genomic DNA isolation, strains were grown for 16 h on tryptic soy agar (TSA) at 37 °C or in tryptic soy broth (TSB) with overnight shaking at 200 r.p.m. at 37 °C, and DNA isolated as described previously (Spoor *et al.*, 2013). For the *in vitro* assays, strains were cultured in brain heart infusion (BHI) overnight at 37 °C, shaking at 200 r.p.m.

### Genome sequencing, mapping assembly and *de novo* genome assembly

Paired-end Illumina sequencing was carried out on a Genome Analyzer IIx, following standard Illumina protocols. Read quality was assessed and genome assembly and variant calling were conducted as described previously (Spoor *et al.*, 2013). Reads were aligned against the reference genome MW2 (GenBank accession number NC_003923), a triple-locus variant of ST97. Core genome was defined as sites shared by all strains included in mapping analysis. Effect of variants were determined using SnpEff 3.0 (Cingolani *et al.*, 2012). Pairwise analysis of SNP density was calculated in 10 000 bp windows across the length of the genome. Prior to *de novo* assembly, low-frequency erroneous reads were removed using Quake 0.3.4 (Kelley *et al.*, 2010) and *de novo* assemblies generated using Velvet 1.2.07 (Zerbino & Birney, 2008) using the VelvetOptimizer.pl script implemented in VelvetOptimizer 2.1.7 (http://bioinformatics.net.au/software.velvetoptimiser.shtml).

### Molecular typing and evolutionary analysis of CDSs

MLST typing was conducted using *in silico* using srrs2 (Inouye *et al.*, 2012) and by interrogating genome sequences for the alleles using blast(Altschul *et al.*, 1990), and querying the sequences against the MLST database (http://saureus.mlst.net/). For comparative analysis of the evolutionary origin of the ST71 genome, three CDSs situated within and three CDSs situated out-with the SNP-dense region were arbitrarily selected for phylogenetic analysis from selected strains listed in Table S1. Nucleotide sequences for each CDS were extracted from *S. aureus* genome sequences using blastn (Altschul *et al.*, 1990), aligned using the clustal w method (Larkin *et al.*, 2007) and neighbour-joining trees constructed using mega 4.0 with 1000 bootstrap replicates (Tamura *et al.*, 2007).

### Core genome phylogenetic analysis

The ST71 consensus sequences were combined with the CC97 consensus sequences determined from a previous study and the core genome redefined (Spoor *et al.*, 2013). Maximum-likelihood phylogenetic trees for the CC97 core genome with and without the predicted recombinant region were reconstructed using RAxML-7.2.6 (Stamatakis, 2006), implementing a GTR model with gamma correction for rate heterogeneity and 1000 bootstrap replicates.

### Recombination detection

For input into recombination detection software, an alignment of the study strains along with selected published ruminant-associated *S. aureus* genomes was created using the progressiveMauve algorithm implemented in Mauve 2.3.1 using default settings (Darling *et al.*, 2010). Locally collinear blocks of at least 1000 bp in length were extracted from the XMFA file using the stripSubsetLCB script distributed with Mauve (at http://darlinglab.org/mauve/snapshots/2015/2015-01-09/linux-x64/), concatenated and converted to a FASTA alignment file format. Recombination detection was performed on this FASTA file using BratNextGen (http://www.helsinki.fi/bsg/software/BRAT-NextGen/) (Marttinen *et al.*, 2012), setting the hyper-parameter α to 1, with a cut-off value of 0.1 within the proportion of shared ancestry tree, conducting 40 iterations within the detecting recombination algorithm and 100 permutation runs for estimating significance. In addition, the rdp2 suite of recombination software programs was employed as described (Martin *et al.*, 2005).

### Comparative genomic analysis of ST71 and ST97 isolates

The population-based *de novo* assembly software Cortex 1.0.5.20 was used to identify variation in gene content among ST71 and ST97 strains (Iqbal *et al.*, 2012). Cortex utilizes coloured de Bruijn graphs to detect variant sequence among bacterial population datasets. The ST71 strains listed in Table S1 were defined as group 1 and the bovine ST97 strains were defined as group 2. Variant sequences that were present in at least one strain of each group, but none of the strains in the comparison groups were identified, then filtered as representative of genotype only if that variant sequence was present in all strains within a group. Identified variant CDSs were annotated using Prokka 1.5.2 (Seemann, 2014) and blastx against the nr GenBank database (Altschul *et al.*, 1990). For each strain, *de novo* assemblies were aligned against *S. aureus* Newbould 305 contig 002 (GenBank accession number AKYW01000002) using the Mauve Contig Mover tool implemented in Mauve 2.3.1 (Darling *et al.*, 2010).

### Western blot analysis of cell envelope components

Exponential phase (OD_600_ 0.6) *S. aureus* cultures were washed in PBS before suspension in 50 mM Tris/HCl, 20 mM MgCl_2_, 30 % raffinose (Sigma-Aldrich), pH 7.5 supplemented with 200 μg lysostaphin ml^− 1^ (AMBI) and protease inhibitors (Roche). After incubation at 37 °C, 20 min and centrifugation at 6000 *g*, 20 min, the cell wall-associated proteins were analysed by Western blot with 3B12 anti-Cna mouse mAb (100 ng ml^− 1^) incubated for 2 h in 1 % milk-PBST (0.5 % Tween-20). After washing, the blot was incubated for 1 h with HRP-conjugated goat F(ab) anti-mouse IgG (1 μg ml^− 1^) (Abcam). Cna-positive *S. aureus* strain ATCC 25923 and Cna-negative *S. aureus* strain Newman were used as controls. Capsule assays were performed as described previously (Luong *et al.*, 2011) using cultures grown in TSB without glucose. Assays for polysaccharide intercellular adhesin (PIA; also known as poly-N-acetylglucosamine) were performed according to the method described previously (Cue *et al.*, 2009).

### Biofilm assay

Biofilms were grown in hydrophilic (Nunclon) 96-well plates, as described previously (Waters *et al.*, 2014). Briefly, overnight cultures were diluted 1 : 200 in either BHI broth, or BHI supplemented with 4 % NaCl or 1 % glucose and grown for 24 h at 37 °C. The plates were then washed three times in distilled water and biofilms stained with 0.5 % crystal violet. Biofilm density was measured at *A*_490_.

### Bacterial adherence to immobilized collagen

Bacterial adherence assays were conducted as described previously (Bannoehr *et al.*, 2011). Microtitre plates were coated with doubling dilutions of commercially available bovine collagen type I (Life Technologies) in PBS at 4 °C for 15 h. After blocking in 4 % milk, exponential phase *S. aureus* cultures were standardized to OD_600_ 1.0 in PBS and applied to the plate. After crystal violet staining for 3 min and 5 % acetic acid treatment the plates were analysed at 590 nm (Synergy HT; BioTek). Each assay was performed in triplicate and each experiment was repeated at least twice independently. Inhibition assays were performed by incubating the standardized *S. aureus* strains in PBS with increasing concentrations of anti-Cna mouse mAb for 1 h at 37 °C before being applied to microtitre plates coated with 1 μg bovine collagen type I ml^− 1^.

### Bovine mammary epithelial cell invasion assays

Bovine mammary epithelial cell line MAC-T was grown in media containing Dulbecco's modified Eagle's medium (DMEM) with 10 % (v/v) FBS (Gibco), 1 % (w/v) penicillin/streptomycin solution (Invitrogen) and 5 μg bovine insulin ml^− 1^ (Sigma), and incubated at 37 °C in a humidified incubator with 5 % CO_2_. Cells were split when confluent with Tryple Express (Invitrogen). For the invasion assays, MAC-T cells were seeded into 24-well plates at 1 × 10^5^ cells per well using the growth media and kept at 37 °C with 5 % CO_2_ until confluent. On the day of the experiment, the wells were washed with warm PBS three times, and media containing only DMEM was then added to the wells and kept at 37 °C with 5 % CO_2_. Bacteria were grown to OD_600_ 0.6 in DMEM and co-cultured with the MAC-T cells for 2 h at m.o.i. 25. The cells were washed with PBS three times and DMEM medium with 150 μg gentamicin ml^− 1^ was added to each well followed by incubation at 37 °C with 5 % CO2 for 30 min to kill any extracellular bacteria. The cells were washed again with PBS and lysed with 0.1 % Triton-X in PBS. The cell lysates were serially diluted, plated on TSA plates and incubated overnight at 37 °C to estimate the number of intracellular bacteria. The percentage invasion was calculated as a ratio of intracellular bacteria compared with the inoculum.

### Cloning, recombinant protein expression, mitogenicity and Vβ specificity assays

For gene cloning, forward primer 5′-TAGCCTCGAGAGACACAAAATGATCCAAA-3′ was designed to amplify within the coding sequence of the *selz* gene from strain RF122 (SAB0026), after the signal peptide predicted by the Signal P 3.0 Server (http://www.cbs.dtu.dk/services/SignalP/). The gene in RF122 has an identical derived amino acid sequence in the mature protein (there is a single synonymous mutation) compared with the RF103 gene. Reverse primer 5′-CGCCTCGAGCTACTTTTTAGTTAAGT-3′ was designed to overlap the stop codon of the gene. *Xho*I sites were incorporated to facilitate cloning into the pET15b plasmid (Novagen). Cloning and recombinant protein purification were carried out as described previously (Wilson *et al.*, 2011). Peripheral blood mononuclear cells were isolated from the blood of Holstein–Friesian cattle aged 18–36 months via jugular vein puncture by density-gradient centrifugation using Ficoll Plaque Plus (GE Healthcare) as described previously (Wilson *et al.*, 2011). rSElZ protein was incubated with 1 × 10^6^ cells ml^− 1^ for 72 h at 37 °C, 5 % CO_2_ in complete cell culture medium (RPMI 1640; Gibco) supplemented with 10 % heat-inactivated FCS, 100 U penicillin ml^− 1^, 100 μg streptomycin ml^− 1^, 292 μg l-glutamine ml^− 1^ (PSG) and 50 μM 2-mercaptoethanol (Sigma-Aldrich). Cells were cultured for a further 18 h after the addition of 1 μCi [^3^H]thymidine. Cellular DNA was harvested onto glass fibre filters and [^3^H]thymidine incorporation was measured by liquid scintillation counting as described previously (Wilson *et al.*, 2011). Total RNA was extracted before and after stimulation with rSElZ (1 μg ml^− 1^) for 96 h. Expansion of cells expressing different bovine Vβ gene subfamilies was determined using quantitative real-time PCR as described previously (Wilson *et al.*, 2011).

## Results

### Identification of a highly divergent subtype of the major livestock-associated *S. aureus* clone CC97

**Fig. 1.**
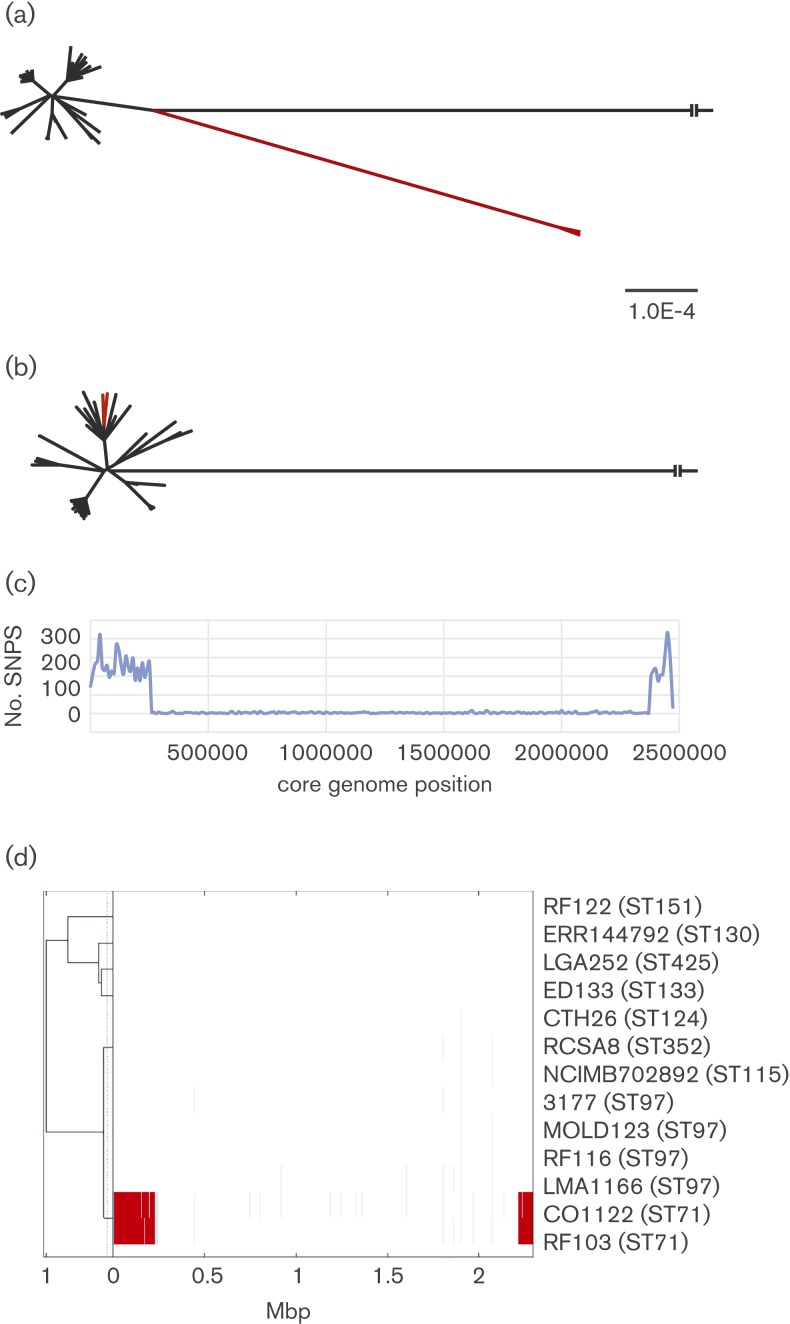
ST71 is a divergent subtype of CC97 containing a 323 kb genomic region with a distinct evolutionary origin. Maximum-likelihood tree constructed from core genome alignment with (a) and without (b) the SNP-dense region. The outgroup is strain MW2; ST71 strains are highlighted in red. CC97 strains included are those listed in Table S1 in addition to the ST71 strains. Scale is indicated for each tree in substitutions per site. (c) Pairwise strain comparisons of core genome SNP density over 10 000 bp windows between ST71 strain RF103 and ST97 strain RF116 (blue line). (d) Identification of multiple large-scale recombination events in the ST71 genome. The names of each strain are shown on the right with MLST sequence genotype indicated in parentheses. On the left side of the diagram is the proportion of shared ancestry tree as determined by BratNextGen (Marttinen *et al.*, 2012). The coloured region indicates detected recombination events along the length of the core genome alignment. The same colour at overlapping genomic locations in different strains indicates those segments are of the same origin. Faint grey lines indicate alignment gaps.

The *S. aureus* CC97 lineage is a leading cause of bovine mastitis on a global scale (Fitzgerald, 2012; Smyth *et al.*, 2009; Spoor *et al.*, 2013). We previously identified a single-locus variant of ST97 (ST71) among dairy herds in Ireland that had a capsule genotype (capsule type CP8) distinct from other CC97 strains (capsule type CP5) (Guinane *et al.*, 2008). Subsequently, ST71 has been isolated from bovine mastitis in countries in Europe, North America and Asia, indicating successful inter-continental dissemination (Guinane *et al.*, 2008; Ikawaty *et al.*, 2009; Li *et al.*, 2015; Smith *et al.*, 2005, 2009). Here, in order to examine the evolutionary history of the ST71 clone and to examine the molecular basis for the switch in capsule serotype, we carried out comparative whole-genome sequence analysis of ST71 with other CC97 isolates and reconstructed the CC97 phylogeny using a maximum-likelihood approach. Strikingly, the ST71 isolates were highly divergent and clustered together at the tips of a long branch relative to all other CC97 isolates, implying either recombination or an elevated mutation rate for ST71 (Fig. 1a). Network analysis of the extracted SNP alignment using the program SplitsTree (Huson & Bryant, 2006) revealed evidence of a reticulate structure at the junction of the bovine ST97 and ST71 strains (Fig. S1), with the ϕ test providing strong statistical support for recombination (*P* = 1.154 × 10^–7^). Comparison of whole-genome sequences of isolates RF103 and RF116 representing ST71 and ST97, respectively, by mapping to a reference genome (MW2) revealed a core genome alignment of 2 472 592 nt with 4905 SNPs in total (Table 1). Of note, 4463 (91 %) of the SNPs were located within an ∼330 kb (SNP-dense) region spanning the origin of replication (*oriC*) starting within a gene encoding fructose 1,6-bisphosphonate aldolase and ending within a gene encoding a hypothetical protein, representing ∼15 % of the chromosome, with only 443 SNPs (9 % of the total) identified out-with this region (Figs. 1c and S2, Table 1). It was noteworthy that the SNP-dense region contained a higher proportion of synonymous to non-synonymous mutation (62 % : 21 %) compared with the rest of the genome (25 % : 52 %), consistent with a distinct evolutionary history (Table 1) (Castillo-Ramírez *et al.*, 2011). Strikingly, masking of the SNP-dense region in the CC97 alignment resulted in a phylogeny with a markedly reduced ST71 branch length, consistent with the recent shared ancestry of ST71 and CC97 (Figs. 1b and S1).

**Table 1.** Core genome sequence diversity between ST71 (RF103) and ST97 (RF116)

Type of SNP	SNPs [*n* (%)]			
	Within SNP-dense region		Out-with SNP-dense region	
Non-synonymous SNPs	936	(21)	229	(51.6)
Synonymous SNPs	2766	(62)	108	(24.5)
Intergenic	749	(16.8)	99	(22.3)
Other effects (strain affected)*	1 × StL	(RF116)	1 × StL	(RF116)
	5 × SG	(RF103)	3 × SG	(RF103)
	3 × SG	(RF116)	3 × SG	(RF116)
	1 × SpL	(RF116)		
	1 × SpL	(RF103)		
Total SNPs	4462		443	

* Other effects: loss of a start codon (StL), gain of a stop codon (SG), loss of a stop codon (SpL).

In order to examine the evolutionary history of the SNP-dense region within the *S. aureus* ST71 genome, we reconstructed the phylogeny of selected CDSs located within (*n* = 3) or out-with (*n* = 3) the SNP-dense region (Fig. S3). For each gene tree, the CDSs located within the SNP-dense region of the ST71 *S. aureus* strains was more closely related to non-ST97 than to ST97 sequences, while the CDSs out-with the SNP-dense region co-segregated with ST97 alleles (Fig. S3). Taken together, the phylogenetic and sequence analysis data implied that ST71 has a hybrid genome that contains an ∼330 kb region with an evolutionary history distinct from that of the ancestral ST97.

### Recombination events have led to the mosaic genome of ST71 *S. aureus*

**Fig. 2.**
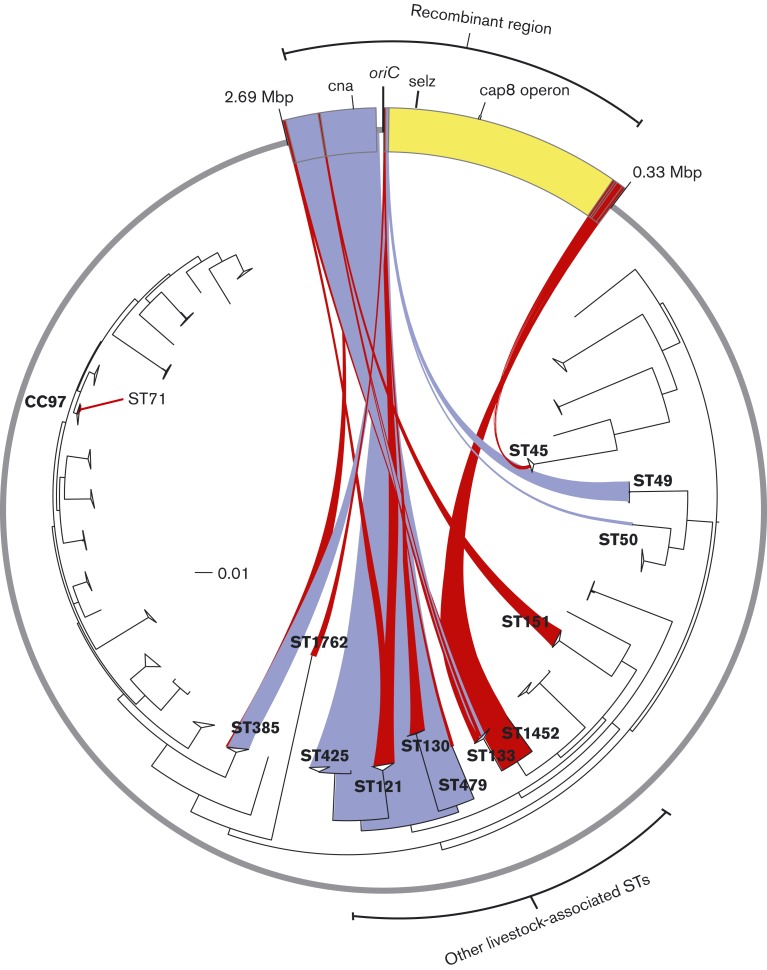
Tracing the evolutionary origin of ST71 recombinant sequences. The circle represents the whole-genome sequence of *S. aureus* ST71, with the 10 predicted recombinant regions spanning the origin of replication indicated. Inside the circle is a neighbour-joining phylogenetic tree of 178 isolates representing the full breadth of *S. aureus* species diversity. Filled blue and red lines connect each predicted recombinant region with its likely clonal origin across the *S. aureus* species, based on sequence identity. Red and blue denote a minimum 99.75 and 99.5 % shared non-variant sites, respectively. Yellow depicts the recombinant region which did not share nucleotide sequence identity of at least 99.5 % with any sequence in the database. The locations of selected acquired genes are indicated. The scale represents substitutions per site.

To investigate the molecular events which led to the hybrid genome of ST71, we constructed a core genome alignment of the CC97 isolates (including ST97, ST71 and other single-locus variants), and selected non-CC97 ruminant *S. aureus* strains, and employed the recombination detection software BratNextGen in order to identify predicted recombination events, breakpoints and potential donor sequences (Fig. 1d) (Marttinen *et al.*, 2012). A region of ∼330 kb in the ST71 chromosome that correlates with the SNP-dense region was predicted to be the result of multiple recombination events (Fig. 1d). In total, 10 predicted recombinant segments were identified in both ST71 strains RF103 and CO1122 (Tables 2 and S2). Similar recombinant regions were identified using the rdp2 suite of recombination detection programs (Martin *et al.*, 2005). There was no evidence of recombination events among the non-CC97 ruminant-associated clones included in the analysis (Fig. 1d). Alignment of each recombinant sequence with genome sequences from a database of 178 *S. aureus* genome sequences selected to broadly represent the diversity across the species revealed that the majority of ST71 recombinant sequences were most closely related to sequences from a small number of ruminant *S. aureus* clonal lineages (Table 2, Fig. 2). However, the largest estimated recombination fragment of 224 kb was not closely related to any clonal complex, implying it originated in an unsampled or extinct strain (Fig. 2). Most of the predicted recombinant regions were contiguous, but three intervening regions (IR1 to IR3) were identified which comprised of 29, 22 and 9046 nt, respectively (Tables 2 and S2). It is feasible that IR3 also represents an additional recombinant fragment as it contained sequence not closely related to ST97, but it was not detected as such by the recombination detection programs employed. Taken together, the recombination detection analysis suggests multiple distinct genetic imports into an ST97 progenitor. However, the collinearity of the predicted recombination region and the lack of any vestigial ST97-like sequences identified between predicted recombinant fragments means that a single large import from a currently unsampled or extinct *S. aureus* genotype cannot be ruled out. Overall, these data indicate that the ST71 clone has emerged through recombination with ruminant-adapted *S. aureus* clones sharing the same environmental niche.

**Table 2.** Estimated recombinant fragment sizes and breakpoints for ST71 strain RF103

Recombinant fragment or intervening region	Start	End	Size (bp)	Sequence types with highest nucleotide identity (CC)*	Nucleotide identity (%)
1	2 689 486	2 692 233	2750	133, 121	99.93
IR1	2 692 233	2 692 262	29	Many	100
2	2 692 262	2 735 451	43 189	481 (133)	99.79
3	2 735 451	2 736 130	679	**151, 522**	100
4	2 736 130	2 792 313	56 183	**479, 2503**	99.65
IR2	2 792 313	19	22	Many	100
5	19	825	807	**2503, 479,** 121	99.88
6	826	3202	2377	**2503, 479**	99.71
7	3203	309 062	268 237	None	
8	309 063	311 660	2598	45	100
IR3	311 661	320 707	9046	None	
9	320 707	326 130	5423	45	100
10	326 130	328 433	2303	**133, 425,** 45	100

* Sequence types predominantly associated with ruminants are indicated in bold. Minimum threshold is 99.5 % shared non-variant sites (Fig. 2).

### Recombination-associated diversification of the gene complement mediating host–pathogen interactions

In order to investigate the potential impact of the recombination events on ST71 emergence and niche adaptation, we examined the presence, absence and allelic variation of genes located in the recombinant region of ST71 in comparison with the ancestral ST97 genetic background. Our analysis revealed that the horizontal import of large genome fragments from other ruminant-associated lineages resulted in a diversification of gene content marked by loss of at least 44 genes and gain of nine genes compared with the ancestral ST97 (Table 3). Of note, many of the variable genes were predicted to influence host–pathogen interactions. For example, the 7 kb operon required for histidine biosynthesis was absent in ST71. The histidine biosynthesis operon was strongly upregulated during human nasal colonization, but its absence indicated its dispensability for ST71 survival in the bovine niche (Krismer *et al.*, 2014). Of note, previous studies of *Lactococcus lactis* and *Streptococcus thermophilus* isolates from dairy sources reported a common phenotype of histidine auxotrophy which evolved through frequent independent loss-of-function mutations in the histidine operon (Delorme *et al.*, 1993; Hols *et al.*, 2005). These data suggest that loss of capacity for histidine biosynthesis may be a specific bacterial adaptation to dairy cows or their environment.

**Table 3.** Variation in gene content associated with the recombinant region of the ST71 genome

Gene	Locus tag *	Product
Genes acquired in ST71 strains		
*cna*	SARLGA251_24600	Collagen adhesin precursor
	SARLGA251_02280	Nitric oxide reductase subunit B
*cap8H*	MW0131	Capsular polysaccharide synthesis enzyme Cap8H
*cap8I*	MW0132	Capsular polysaccharide synthesis enzyme
*cap8J*	MW0133	Capsular polysaccharide synthesis enzyme Cap8J
*cap8K*	MW0134	Capsular polysaccharide synthesis enzyme CapK
*selz*	SAB0026	Staphylococcal enterotoxin-like protein (*selz*)
	MW0064	LysR family transcriptional regulator
	SARLGA251_24290	Putative lipoprotein
Genes lost in ST71 strains		
*cap5H*	Newbould305_0696	Capsular polysaccharide synthesis protein *O*-acetyltransferase Cap5H
*cap5I*	Newbould305_0697	Capsular polysaccharide biosynthesis protein Cap5I
*cap5J*	Newbould305_0698	Capsular polysaccharide synthesis protein Cap5J
*cap5K*	Newbould305_0699	Capsular polysaccharide biosynthesis protein Cap5K
*hsdM*	Newbould305_0618	Type I restriction-modification system DNA methylase
*hsdS*	Newbould305_0619	Type I restriction-modification system specificity protein
*hsdR*	Newbould305_0620	Type I site-specific DNase, HsdR family
*hisD*	Newbould305_0552	Histidinol dehydrogenase
*hisC*	Newbould305_0551	Histidinol phosphate aminotransferase
*hisF*	Newbould305_0547	Imidazole glycerol phosphate synthase subunit HisF
*his1E*	Newbould305_0546	Histidine biosynthesis bifunctional protein HisIE
*hisH*	Newbould305_0549	Imidazole glycerol phosphate synthase subunit HisH
*icaA*	Newbould305_0539	Intercellular adhesion protein A
*icaD*	Newbould305_0540	Intercellular adhesion protein D
*icaC*	Newbould305_0542	Intercellular adhesion protein C
*icaB*	Newbould305_0541	Polysaccharide intercellular adhesin deacetylase icaB
*icaR*	Newbould305_0538	Biofilm operon *icaABCD* HTH-type negative transcriptional regulator IcaR
*sasD*	Newbould305_0674	Cell wall surface anchor family protein
	Newbould305_0560	DNA-directed RNA polymerase subunit delta
*hisA*	Newbould305_0548	1-(5-Phosphoribosyl)-5-(5-phosphoribosylamino) methylideneaminoimidazole-4-carboxamide isomerase
*hisB*	Newbould305_0550	Imidazoleglycerol phosphate dehydratase
	Newbould305_0616	Guanylate cyclase
*hisZ*	Newbould305_0554	ATP phosphoribosyl transferase regulatory subunit
	Newbould305_0555	Polysaccharide deacetylase
	Newbould305_0480	Metallo-β-lactamase
	Newbould305_0633	ATPase
	Newbould305_0558	Cobalt ABC transporter ATP-binding protein
	Newbould305_0544	Lipase
	Newbould305_0633	RNA helicase
	Newbould305_0638	Tandem lipoprotein
	Newbould305_0562	Lactonase Drp35
	Newbould305_0533	Methionine sulfoxide reductase A
	Newbould305_0555	Polysaccharide deacetylase
	Newbould305_0632	Membrane spanning protein
	Newbould305_0563	Rhodanese domain sulfur transferase
	Newbould305_0534	Acetyltransferase
	Newbould305_0564	Pyrrolidone carboxylate peptidase
	Newbould305_0641	Amidohydrolase
*cysG*	Newbould305_0486	Precorrin-2 dehydrogenase
	Newbould305_0793	Hexitol dehydrogenase
	Newbould305_0736	RND transporter
	Newbould305_0757	NADH-dependent dehydrogenase
	Newbould305_0815	Ribose transporter RbsU
	Newbould305_0748	γ-Glutamyltransferase
	Newbould305_0719	4′-Phosphopantetheinyl transferase

* Locus tags according to annotations in strain MW2 (GenBank accession number NC_003923); in the case of core variable genes that are not present in MW2, alternative locus tags from bovine strains RF122 (GenBank accession number NC_007622) and LGA251 (GenBank accession number FR821779) are listed.

Through recombination-mediated replacement, ST71 has lost the gene encoding the cell wall-anchored protein SasD, in addition to the entire *ica* operon (*icaABCD* operon and transcriptional repressor *icaR*), responsible for the biosynthesis of PIA, an essential component of polysaccharide-mediated biofilm formation (O'Gara, 2007). Lack of production of PIA by ST71 strains in comparison with *ica*-positive ST97 strains was confirmed by Western blot analysis with PIA-specific sera (Fig. S4), indicating that a polysaccharide-dependent biofilm is not required in the niche occupied by ST71 (O'Gara, 2007). We carried out a biofilm assay in the presence of NaCl or glucose which would support the production of a polysaccharide or protein-based biofilm, respectively. Considerable variation in the capacity to produce biofilm was observed among CC97 strains (Fig. S5). Of note, neither ST71 strain produced a biofilm in the presence of NaCl, consistent with lack of a PIA biosynthetic operon. However, one of the ST71 strains produced biofilm in the presence of glucose, demonstrating the capacity for production of a protein-based biofilm (Fig. S5).

Previously, by whole-genome microarray and PCR, we identified a capsule serotype genetic difference between ST71 and ST97 strains (Guinane *et al.*, 2008). Here, we confirmed that ST71 has undergone a recombination-mediated capsule serotype gene switch from CP5 to CP8. Expression of CP5 and CP8 by selected ST71 and ST97 strains was examined by Western blot analysis. Several strains did not express detectable levels of capsule, consistent with the high prevalence of non-capsular bovine *S. aureus* evolved through loss-of-function mutations in capsular biosynthesis genes (Cocchiaro *et al.*, 2006). One of the ST97 isolates examined was strongly positive for CP5 in contrast to both ST71 strains, consistent with loss of CP5 capsule serotype genes. However, considerable cross-reactivity was observed for the CP8-serotype antibody among CP8 gene-negative strains, limiting the capacity to draw conclusions regarding the gain of CP8 expression by ST71 (Fig S4). Capsule serotype switching is widely reported for *Streptococcus pneumoniae* strains and reported to be the result of selection for humoral immune evasion, particularly in the context of vaccine escape (Croucher *et al.*, 2011, 2013, 2015; Wyres *et al.*, 2015). Recent studies of *Streptococcus pneumoniae* and the *Klebsiella pneumonia* hybrid clone ST258 suggest that macro-recombination events may be driven, at least in part, by immune selection for a switch in capsule type (DeLeo *et al.*, 2014; Wyres *et al.*, 2015). To the best of our knowledge, this is the first identification of a recombination-mediated serotype switch for *S. aureus*.

In addition to gene loss or replacement events, the import of large chromosomal fragments from other lineages into ST71 has resulted in acquisition of genes encoding proteins involved in host–pathogen interactions, including a gene encoding nitric oxide reductase which may enhance survival within macrophages in response to the bactericidal killing activity of nitric monoxide radicals (NO^− ^). Furthermore, ST71 has gained genes for a novel lipoprotein, novel putative superantigen and Cna, a cell wall-anchored protein that mediates binding to collagen and inhibition of the classical activation pathway of the complement system (Kang *et al.*, 2013). Taken together, the large-scale recombination events of ST71 resulted in extensive diversification of the complement of genes which would influence host–pathogen interactions.

### ST71 has acquired new pathogenic traits associated with bovine immunomodulation and extracellular matrix interactions

**Fig. 3.**
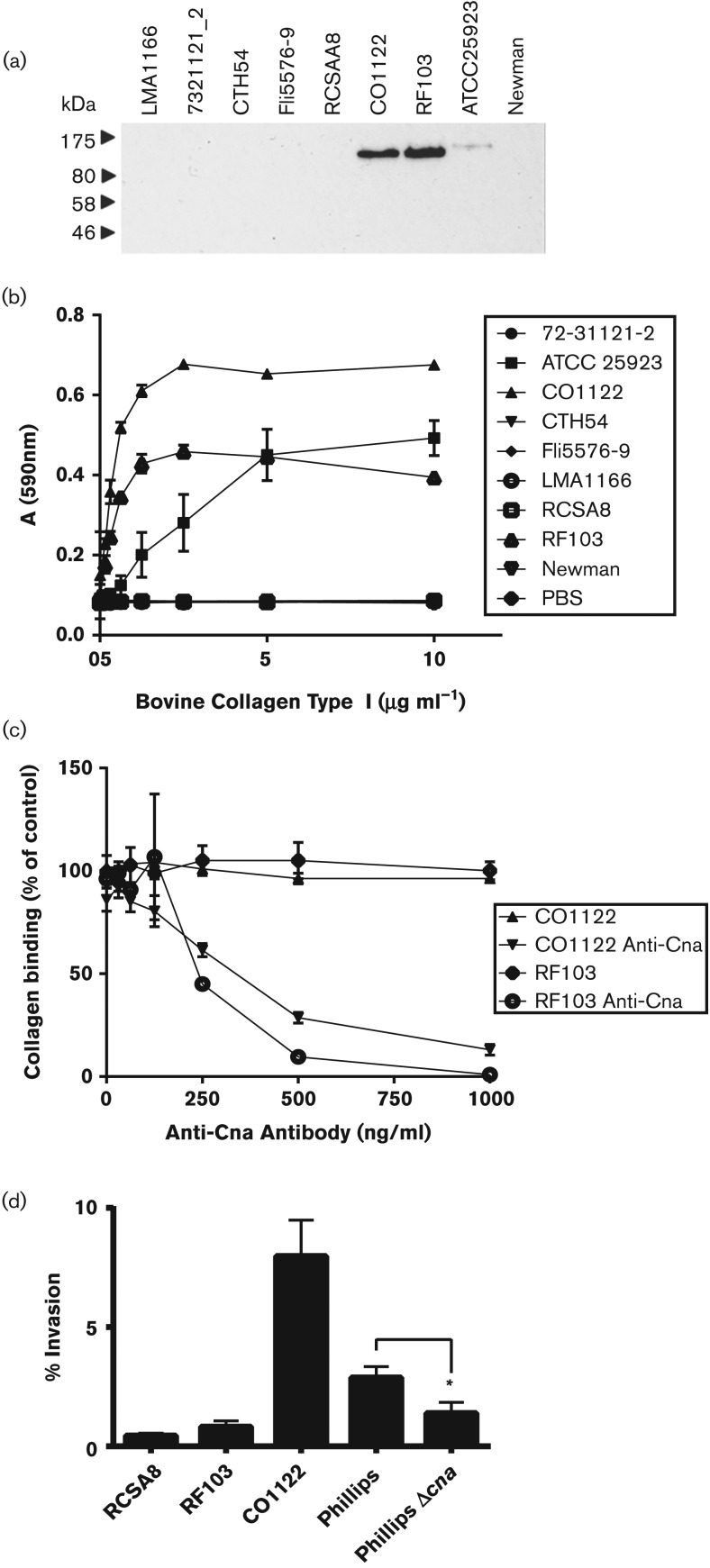
Acquisition of *cna* by ST71 confers the capacity to bind bovine type I collagen. (a) Cna is expressed on the surface of ST71 strains. Western blot analysis of cell wall-associated proteins of ST71 and ST97 strains with anti-Cna mouse mAbs. *S. aureus* ATCC 25923 was used as a positive control and *S. aureus* Newman was used as a negative control. (b) ST71 strains bind to immobilized type I collagen. Plates were coated with doubling dilutions of bovine collagen type I and incubated with *S. aureus* cultured to exponential phase. Results are expressed as mean ± sd *A*_590_ values of triplicate results. *S. aureus* strain ATCC 25923 and strain Newman were used as controls. (c) ST71 Collagen binding is inhibited by anti-Cna antibodies. Exponential phase *S. aureus* were pre-incubated with anti-Cna antibody before addition to the plate. Results are expressed as mean ± sd *A*_590_ values of triplicate results. *S. aureus* strain ATCC 25923 and strain Newman were used as controls (data not shown). (d) Invasion of bovine mammary epithelial cells (MAC-T) by *S. aureus*. Bacteria were co-cultured with MAC-T cells for 2 h at 37 °C, followed by addition of gentamicin to deplete extracellular bacteria. Percentage invasion was estimated by measuring viable counts after cell lysis compared with initial inoculum. Data represent mean ± sem of at least four independent experiments. The reduction in invasion between *S. aureus* Phillips and Phillips Δ*cna* was statistically significant using the Mann–Whitney test (*P* = 0.014).

In order to investigate the potential impact of recombination-mediated gene acquisition by ST71 on ecological success and host adaptation, we examined the phenotypic consequences of selected imported genes implicated in pathogenesis. First, we investigated the functional effect of the import by recombination of the gene (*cna*) encoding Cna on the interaction of ST71 with the bovine extracellular matrix and intra-mammary epithelium. The *cna* gene has been reported to be present at a higher frequency in bovine strains of *S. aureus* compared with human clinical isolates, but the role of Cna in pathogenesis of the bovine udder has not been investigated (Delgado *et al.*, 2011; van Leeuwen *et al.*, 2005). Expression of Cna by *S. aureus* ST71 isolates, and lack of expression by ST97 isolates, was demonstrated by Western blot analysis with a Cna-specific mAb (Fig. 3a). Furthermore, adherence assays revealed that ST71 isolates, but not ST97 isolates, had the capacity to adhere to immobilized bovine type I collagen and this binding activity was reduced in a dose-dependent manner by pre-incubation with the Cna-specific mAb (Fig. 3b, c). A previous study of bovine *Streptococcus uberis* reported a role for collagen binding in bovine mammary epithelial cell interactions (Almeida *et al.*, 1999). We measured the capacity for ST71 strains to invade bovine mammary epithelial cells (MAC-T) *in vitro* and found a strain-dependent invasion capacity that did not correlate with the abundance of Cna in cell wall fractions detected by Western blot analysis (Fig. 3d). Of note, a previous study highlighted the role of capsular polysaccharide in masking Cna expression and binding activity *in vitro* (Gillaspy *et al.*, 1998), consistent with the capsule-negative, higher collagen-binding phenotype of strain C01122 compared with the capsule-positive, lower collagen-binding activity of RF103 observed in the current study (Figs. 3d and S4). As a control in the invasion assay, we measured the ability of *S. aureus* strain Phillips and its isogenic mutant deficient in Cna expression to invade bovine mammary epithelial cells (Patti *et al.*, 1994). In contrast to the parental strain Phillips, the *cna* isogenic mutant demonstrated a significant decrease in invasion, supporting a role for Cna in promotion of bovine epithelial cell invasion (Fig. 3d). In summary, molecular functional analysis has demonstrated that recombination-mediated acquisition of the *cna* gene has resulted in a gain of function by ST71 strains facilitating adherence to bovine collagen, the major protein component in bovine mammary tissue. Furthermore, the previous report that Cna can bind to the collagenous domain of C1q and interfere with complement activation suggests that acquisition of Cna may also confer an innate immune evasion strategy (Kang *et al.*, 2013). Additional experimental work is required to elucidate the role of Cna in *S. aureus*–epithelial cell interactions.

**Fig. 4.**
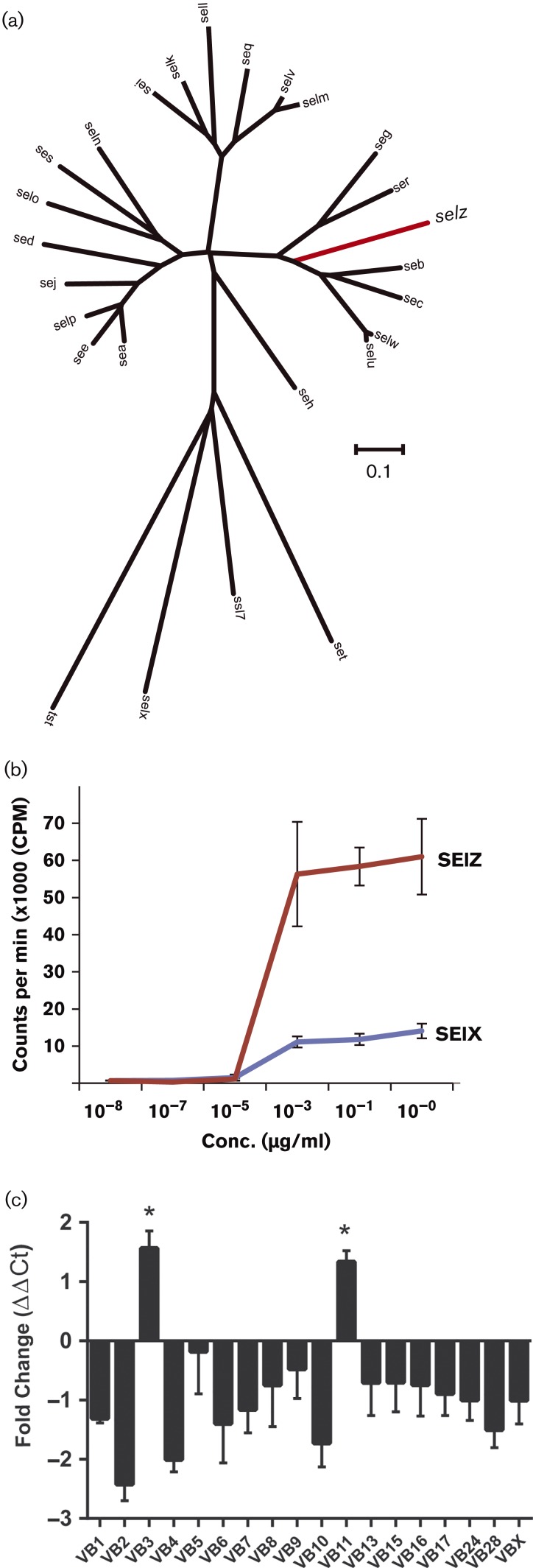
SElZ is a novel superantigen acquired by ST71 which stimulates proliferation of specific bovine T-cell subfamilies. (a) Neighbour*-*joining tree based on the nucleotide sequences of all known staphylococcal superantigens and the superantigen-like protein SSL7. The branch specific for the novel superantigen SElZ is indicated in red. The scale represents substitutions per site. (b) Proliferation of bovine peripheral blood mononuclear cell cultures in response to recombinant SElZ (red) and SElX (blue) measured by [^3^H]thymidine incorporation. (c) Relative fold change in Vβ expression (mean ± sem) for bovine T-cells from two donors after stimulation with SElZ in triplicate. *Statistical significance for Vβ3 (*P* = 0.029) and Vβ11 (*P* = 0.05).

Secondly, ST71 has acquired the gene for a putative novel superantigen, that we named staphylococcal enterotoxin-like toxin Z (*selz*). Superantigens contribute to disease pathogenesis by activating specific subpopulations of T-cells, resulting in T-cell anergy and dis-regulation of the acquired immune response (Spaulding *et al.*, 2013). In particular, bovine isolates typically have multiple genes encoding superantigens that demonstrate host-specific activity, suggesting an important role in host adaptation (Deringer *et al.*, 1997; Fitzgerald *et al.*, 2001). SElZ belongs to the superantigen phylogenetic group and has 65 % amino acid identity with its closest homologue SEG (Fig. 4a). We purified recombinant SElZ and demonstrated that SElZ had dose-dependent mitogenic activity for bovine T-cells (Fig. 4b). Furthermore, we identified that bovine T-cell subpopulations with Vβ3 and Vβ11 receptors were preferentially activated, indicating that SElZ is a novel bovine superantigen (Fig. 4c). These data indicate that recombination-mediated acquisition of the *selz* gene by ST71 enhanced the capacity for non-specific stimulation of bovine T-cell subpopulations, suggesting a role in modulation of the acquired bovine T-cell immune response.

Taken together, the genomic and molecular functional analyses indicate that large-scale recombination events that shaped the evolution of ST71 have resulted in loss, acquisition and allele replacement of an array of pathogenic traits enhancing the capacity of *S. aureus* to survive in the bovine host. Our findings support the idea that large-scale recombination events can have a profound influence on the success of emergent bacterial clones.

## Discussion

**Fig. 5.**
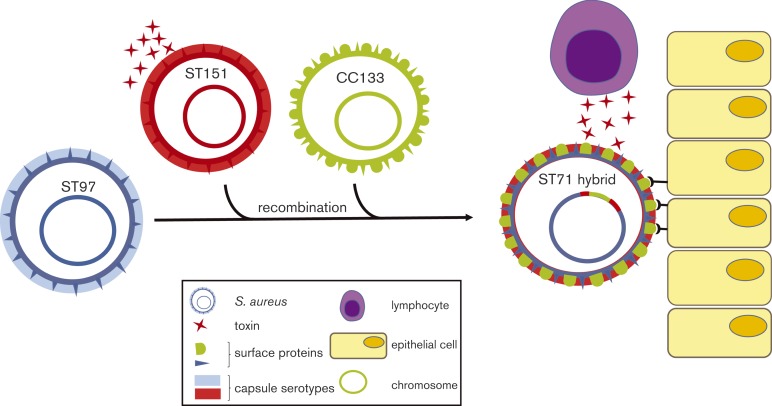
Schematic representation of the evolution and pathogenic diversification of *S. aureus* ST71.

We propose a model for the emergence of the ST71 clone that involved large-scale recombination events influencing host–pathogen interactions and ecological success (Fig. 5). The role of homologous recombination in adaptation of bacteria to different environments is not well understood. Previously, Sheppard *et al.* (2013) reported introgression in a specific *Campylobacter coli* clone with sequences from *Campylobacter jejuni* occupying the same agricultural niche, via genome-wide homologous recombination. The authors highlighted the acquisition of genes involved in the transport and metabolism of fucose, a major component of mucin, and speculated that this may confer a survival advantage in the gut (Sheppard *et al.*, 2013). In contrast to the introgressed *C. coli* clone that was the result of genome-wide homologous recombination of short DNA sequences, the hybrid *S. aureus* clone ST71 was the result of multiple large-scale recombination events affecting a region representing ∼15 % of the genome. To date, several hybrid clones of *S. aureus* have been identified, but the mechanism of import of large chromosomal fragments leading to recombination is unclear. *S. aureus* pandemic clone ST239 was the result of a single genetic import from a ST30 donor lineage into an ST8 background, and hybrid clones ST34 and ST42 also likely evolved from single large-scale recombination events (Robinson & Enright, 2004). It is speculated that these events may have been promoted by conjugative transfer of large chromosomal fragments (Robinson & Enright, 2004). However, some of the smaller predicted recombination events in ST71 could alternatively have arisen by phage-mediated transduction or pathogenicity island-mediated transfer (Chen *et al.*, 2015; Moon *et al.*, 2015). Pan-genus analysis of the *Staphylococcus* genome highlights the plasticity of the region spanning *oriC* (*oriC environ*) in different staphylococcal species, in contrast to the synteny observed across the rest of the genome. The *oriC environ* appears to be a hotspot for recombination across the *Staphylococcus* genus, suggesting a role in ecological specialization of different species (Takeuchi *et al.*, 2005).

It is noteworthy that one of the differences in gene content effected by the recombination observed in ST71 is the replacement of genes encoding a type I restriction modification system with genes for a transposase and five hypothetical proteins (Fig. S6). We speculate that loss of a restriction barrier may support the capacity of *S. aureus* strains to import foreign DNA sequences leading to recombination. However, additional experimental work would be required to test this hypothesis. The apparent enrichment of synonymous SNPs in this region of the genome in ST71 compared with the ST97 background is consistent with the previous observation by Feil and colleagues that non-core and core sequences affected by recombination have lower ratios of non-synonymous to synonymous substitutions reflecting the longer time-frame for purifying selection to purge mildly deleterious mutations (Castillo-Ramírez *et al.*, 2011). Importantly, the CC97 lineage is predicted to have diverged from a human-associated ancestor ∼1200 years ago, making it considerably younger than several of the predicted donors of the recombinant region in ST71, including CC133 and ST151, that are estimated to have originated in humans ∼5400 and ∼3000 years ago, respectively (Weinert *et al.*, 2012). It is reasonable to infer that the import of genes from *S. aureus* donor clones which have been under ruminant host-adaptive selection for several thousand more years could enhance the fitness of the younger recipient clone in the bovine host. The predicted function of the genes affected and the phenotypic effects observed in the current study, including immunomodulation, enhanced adherence and intracellular invasion, lead us to speculate that the ST71 clone may be evolving towards a less pathogenic association with the bovine host. Of note, we previously identified very low levels of RNAIII expression among ST71 strains *in vitro* and reduced bacterial burden relative to ST97 after experimental murine infection, consistent with reduced virulence (Guinane *et al.*, 2008). The possibility that some bovine *S. aureus* strains may be evolving towards a more intracellular lifestyle has been proposed previously (Herron-Olson *et al.*, 2007) and may reflect increased antibiotic selective pressures in the dairy industry driving the bacteria into niches which are less accessible to most antibiotic classes. This may also explain the apparent paucity of resistance determinants among most *S. aureus* isolates from bovine sources (Spoor *et al.*, 2013). However, the very recent emergence of ST71 strains that are meticillin-resistant is a significant cause for concern, and surveillance is required to monitor the dissemination of the ST71 hybrid clone, and to comprehensively assess its threat to veterinary and public health.

Overall, our analysis suggests that the recombination events that shaped the mosaic genome of the ST71 clone have attenuated host–pathogen interactions, enhancing the capacity of *S. aureus* to evade the host immune response and adhere to host tissue. These findings provide a paradigm for the potential impact of large-scale recombination events on the rapid adaptive evolution of bacterial pathogens within defined ecological niches.

## Supplementary Data

1258 Supplementary Data
