# Supplementary material for: Recombination-mediated remodelling of host–pathogen interactions during Staphylococcus aureus niche adaptation
Source: Microb Genom. 2015 Oct 30;1(4):e000036. doi: 10.1099/mgen.0.000036 (PMC5320625; doi:10.1099/mgen.0.000036)
Supplement: Supplementary file 1 — Supplementary Data [file mgen-01-36-s001.pdf]

**a)**

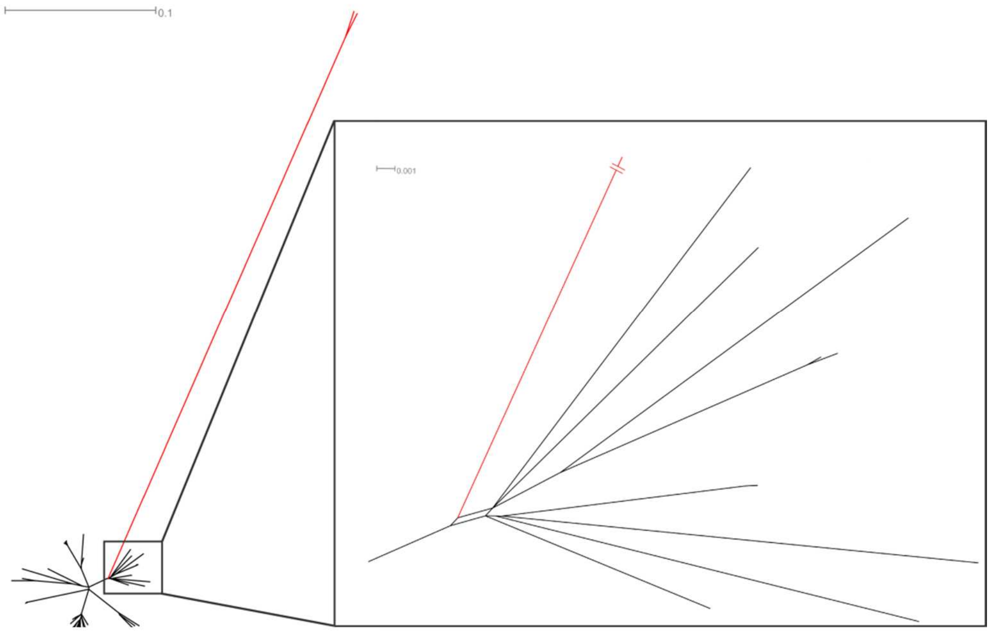


**b)**

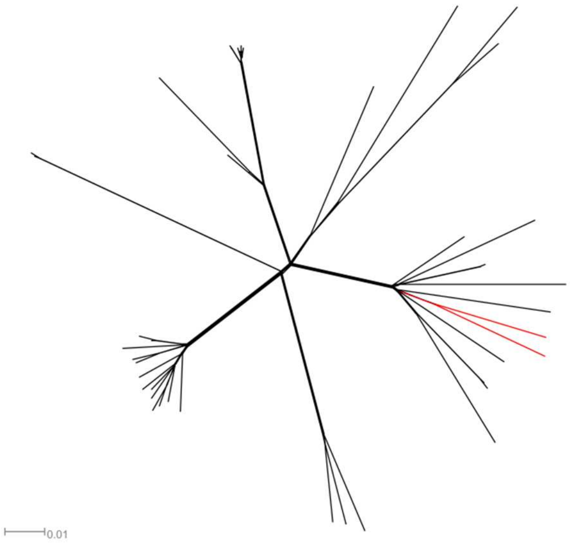


**Fig. S1. Splitstree analysis of core genome SNP alignment for CC97 and ST71.** Phylogeny of ST97 (black branches) and ST71 (red branches) (a) including the SNP-dense region and b) without the SNP-dense region. Diagram a) also includes a magnified view of the nodes between the ST71 strains (red) and the largest clade of bovine CC97 strains (black). Scale bars are as shown.

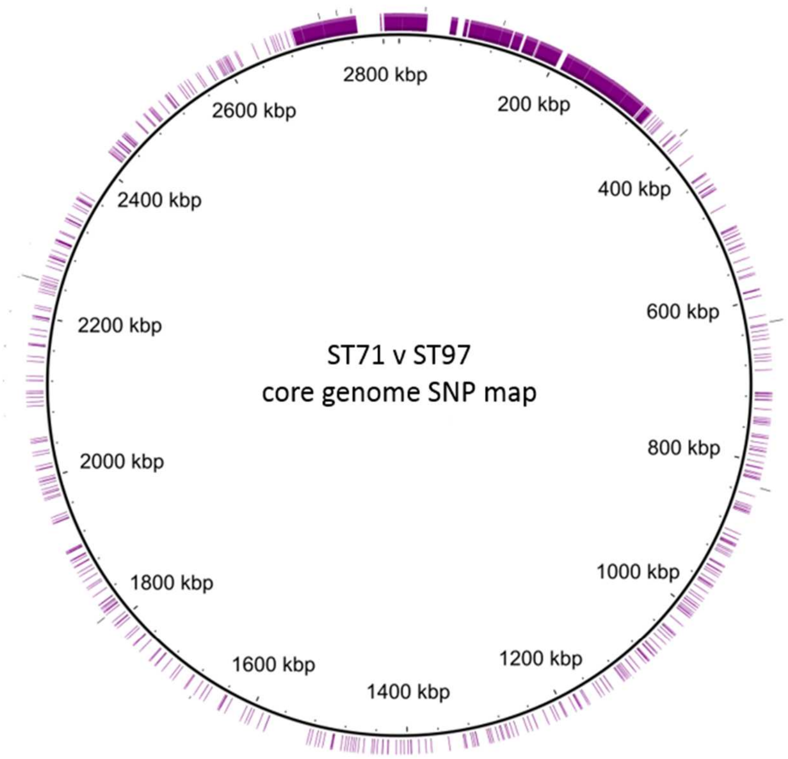


**Fig. S2. Circular diagram illustrating the genome distribution of SNPs between ST71 and ST97.** Purple lines indicate the genome location of SNPs identified between consensus sequences for strains RF103 (ST71) and 38_1993_91 (ST97). Diagram produced using BRIG v0.95 (Alikhan et al, 2011 BMC Genomics 12:402 doi: 10.1186/1471-2164-12-402

a) MW2547 (*phoB*-alkaline phosphatise III precursor)

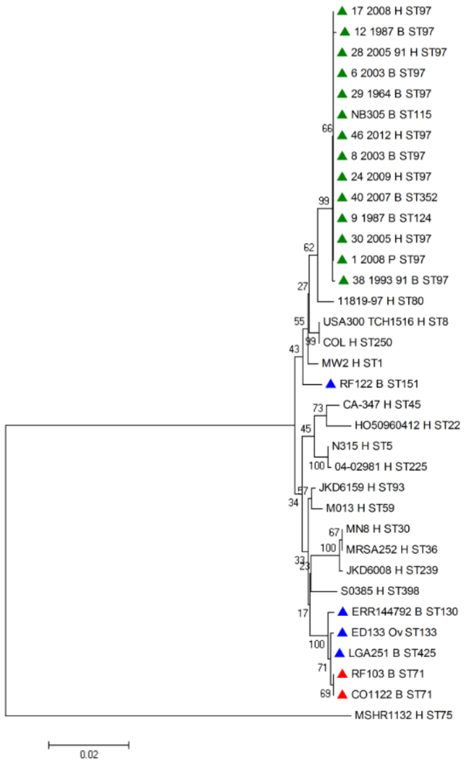


b) MW2611 (Hypothetical protein)

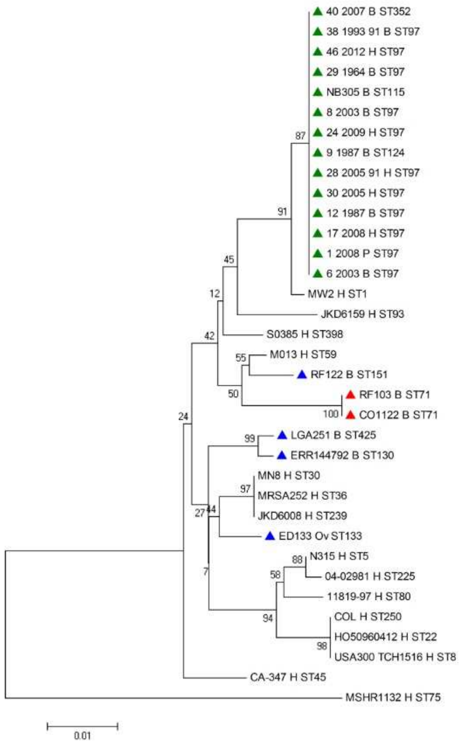


c) MW2630 (*trmE*-tRNA modification GTPase)

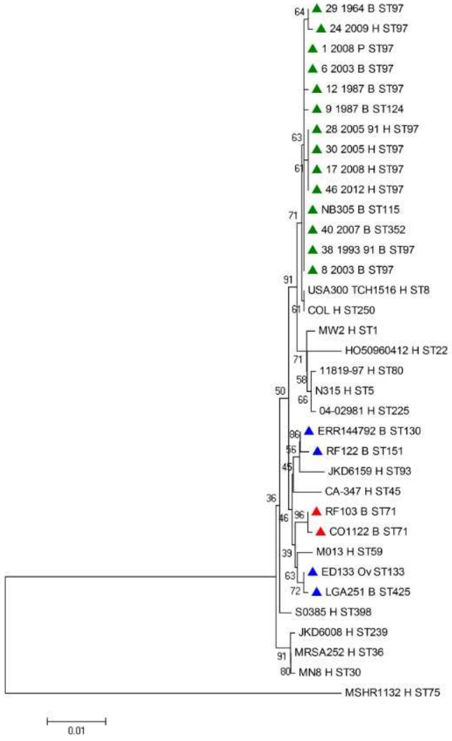

d) MW0365 (*pbuX*-xanthin permease) e) MW0626 (hypothetical protein) f) MW1183(*glpK*-glycerol kinase)

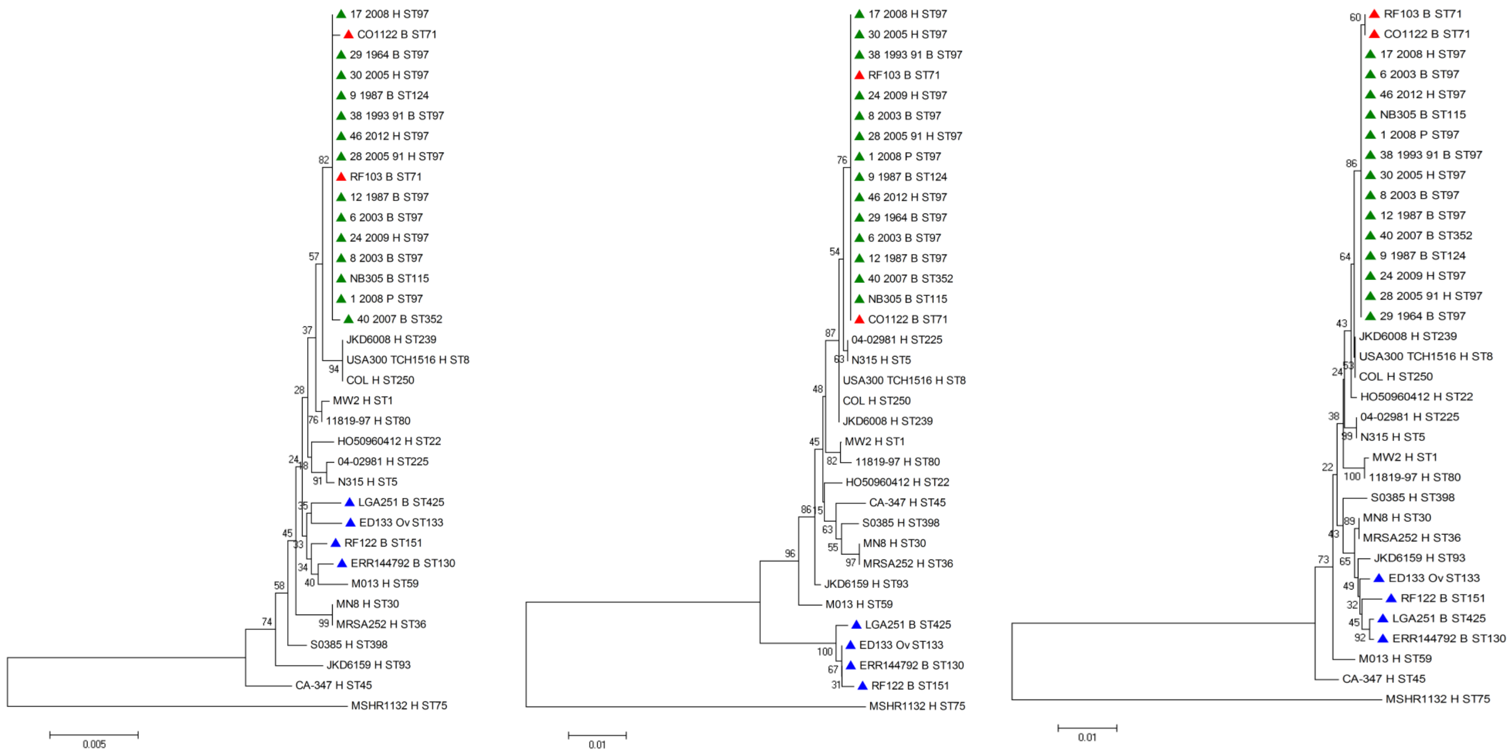


**Fig. S3 Neighbour joining trees for CDS within (a to c) and outwith (d to F) the ST71 SNP-dense region.** Strains are colour-coded according to genotype (red, ST71; green, CC97; blue, non-CC97). Phylogenetic trees produced with 1000 bootstrap replicates, indicated at each node. Taxa are individually labelled according to strain identity, host (B=bovine, H=human, P=pig) and MLST sequence type.

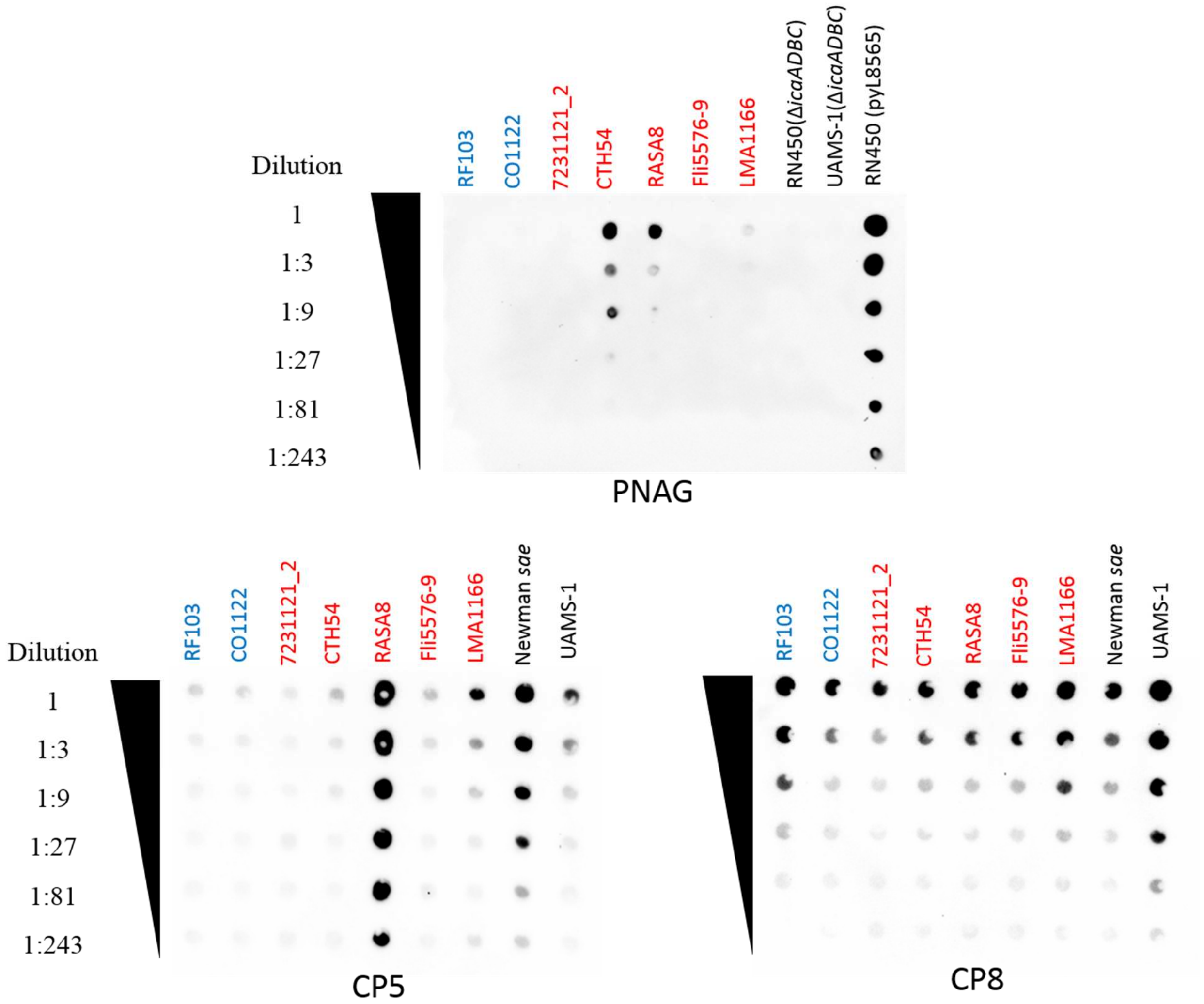


**Fig S4.** Capsule and PIA (PNAG) immunoblot analysis of ST71 (blue text), and ST97 (red text). Newman *sae* is a positive control for CP5 and negative for CP8, and UAMS-1 is positive for CP8, and negative for CP5. RN450 (pyL8565) is a positive control for PNAG, and RN450(ΔicaDBC) and UAMS-1(ΔicaDBC) do not produce PNAG.

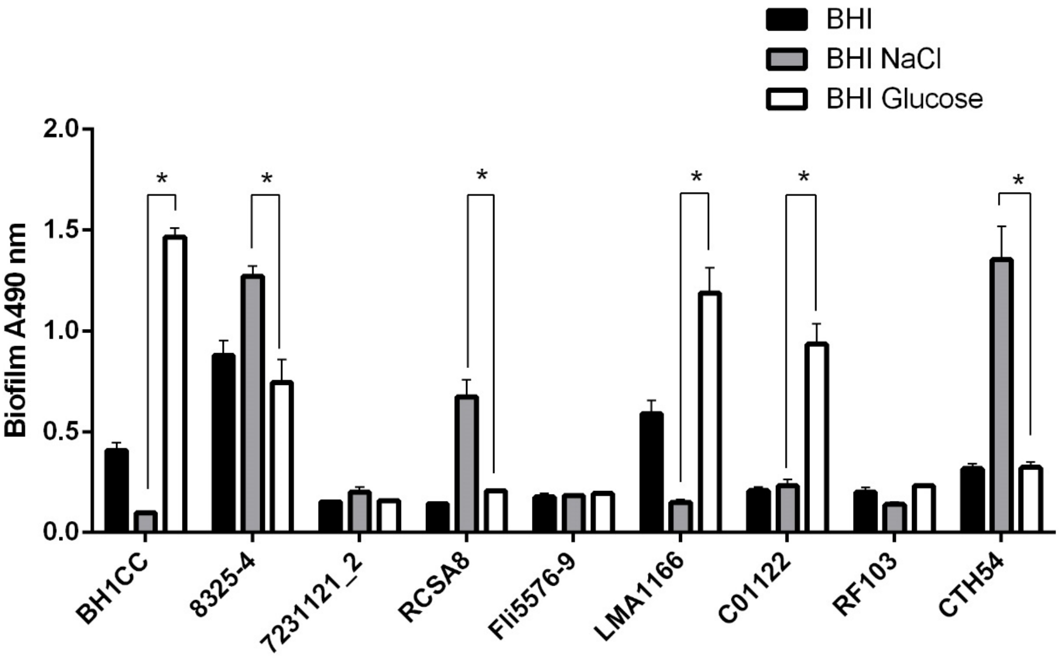


**Fig. S5. Biofilm analysis of CC97 *S. aureus* isolates.** Biofilm formation by *S. aureus* strains BH1CC (MRSA strain that produces FnBP-dependent biofilm in BHI glucose), 8325-4 (laboratory MSSA strain that produces poly-N-acetylglucosamine-dependent biofilm in BHI NaCl), 7231121_2, RCSA8, Fli5576-9, LMA1166, CO1122 (ST71), RF103 (ST71) and CTH54 grown for 24 h on microtiter wells in BHI, BHI supplemented with 4% NaCl or 1% glucose. The density of crystal violet-stained biofilms was measured at $A_{490}$ and the data presented are the means ± standard deviation of 3 independent experiments. *statistically significant.

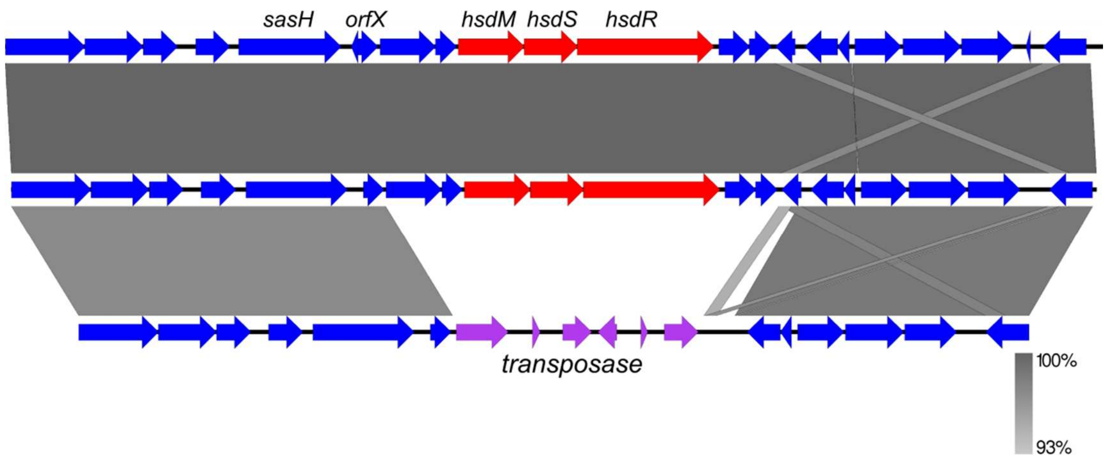


**Fig. S6. Schematic diagram illustrating the region containing the additional type 1 RM system in the ST97 *S. aureus* compared to the ST71 *S. aureus* strains.** Representative strains are as labelled on the left hand side of the diagram. Genes of interest are colour-coded (red=type 1 RM system, purple=putative MGE in RF103).Genes are labelled on the diagram where appropriate. The grayscale illustrates the level of nucleotide identity according to BLASTN pairwise sequence comparison

**Table S1.** ***S. aureus*** **strains and genome sequences employed in this study**

| Strain ID | Country | Host | Sequence type (MLST) | Clonal complex | Accession Number/ Reference |
|---|---|---|---|---|---|
| CO1122 | UK | Bovine | 71 | 97 | Sung et al (2008) Microbiol, 154:1949-59 |
| RF103 | Ireland | Bovine | 71 | 97 | Fitzgerald et al (1997) Epidemiol Infect 119:261-9 |
| Phillips | US | Human | - | - | Patti et al, I&I, 62:152-61 |
| Phillips Δ*cna* | US | Human | - | - | Patti et al, I&I, 62:152-61 |
| ATCC25923 | US | Human | - | - | Holderbaum et al, I&I 1986 4:359–364 |
| Newman | UK | Human | 8 | 8 | AP009351.1 |
| Newman sae | UK | Human | 8 | 8 | Cue et al, (2015) PloS ONE 10:e0123027 |
| RN450(pyL8565) | | - | 8 | 8 | Cue et al (2013) J. Bacteriol 195:1515-24 |
| RN450(ΔicaDBC) | | - | 8 | 8 | Cramton et al (1999) I&I 67:5427-33 |
| UAMS-1 | US | Human | 30 | 30 | Beenkin et al, (2004) J. Bacteriol 186:4665-4684 |
| UAMS-1 (ΔicaDBC) | US | Human | 30 | 30 | Beenkin et al, (2004) J. Bacteriol 186:4665-4684 |
| 12780 | Italy | Porcine | 97 | 97 | PRJEB1411 |
| 7231121_2 | Denmark | Bovine | 97 | 97 | PRJEB1411 |

| | | | | | | |
|---|---|---|---|---|---|---|
| 7807 | Italy | Porcine | 97 | 97 | | PRJEB1411 |
| ALG33 | Algeria | Human | 97 | 97 | | PRJEB1411 |
| CHILE5 | Chile | Bovine | 97 | 97 | | PRJEB1411 |
| CHILE9 | Chile | Bovine | 97 | 97 | | PRJEB1411 |
| CHILE22 | Chile | Bovine | 97 | 97 | | PRJEB1411 |
| CO1899 | UK | Bovine | 97 | 97 | | PRJEB1411 |
| CTH26 | USA | Bovine | 124 | 97 | | PRJEB1411 |
| CTH54 | USA | Bovine | 97 | 97 | | PRJEB1411 |
| CTH163 | USA | Bovine | 97 | 97 | | PRJEB1411 |
| 07-02708 | Turkey | Human | 97 | 97 | | PRJEB1411 |
| 06-01420 | Germany | Human | 97 | 97 | | PRJEB1411 |
| Fli5576-9 | Germany | Bovine | Novel SLV (*glpF*) | 97 | | PRJEB1411 |
| FRA134 | France | Human | Novel SLV (*aroE*) | 97 | | PRJEB1411 |
| H068N | French Guiana | Human | 97 | 97 | | PRJEB1411 |
| H118 | UK | Human | 28 | 97 | | PRJEB1411 |
| HO54060126 | UK | Human | 97 | 97 | | PRJEB1411 |
| HO54120523 | UK | Human | 97 | 97 | | PRJEB1411 |

| | | | | | |
|---|---|---|---|---|---|
| HO64020492 | UK | Human | 97 | 97 | PRJEB1411 |
| HO70420647 | UK | Human | 97 | 97 | PRJEB1411 |
| HO71540648 | UK | Human | 97 | 97 | PRJEB1411 |
| HO91460210 | UK | Human | 97 | 97 | PRJEB1411 |
| HO91740182 | UK | Human | 97 | 97 | PRJEB1411 |
| 3177 | UK | Human | 97 | 97 | PRJEB1411 |
| JVI215 | Denmark | Bovine | 97 | 97 | PRJEB1411 |
| 563 | Brazil | Human | 97 | 97 | PRJEB1411 |
| LMA1166 | France | Bovine | 97 | 97 | PRJEB1411 |
| MOLD123 | Moldova-Chisinau | Human | 97 | 97 | PRJEB1411 |
| KK24 | Turkey | Human | 97 | 97 | PRJEB1411 |
| NCIMB702892 | Canada | Bovine | 115 | 97 | PRJEB1411 |
| NZ189 | New Zealand | Human | 97 | 97 | PRJEB1411 |
| SA5 | Spain | Bovine | 97 | 97 | PRJEB1411 |
| RF111 | Ireland | Bovine | 97 | 97 | PRJEB1411 |
| RF115 | Ireland | Bovine | 97 | 97 | PRJEB1411 |
| RF116 | Ireland | Bovine | 97 | 97 | PRJEB1411 |
| B1020 | UK | Human | 97 | 97 | PRJEB1411 |

| | | | | | |
|---|---|---|---|---|---|
| RCSA8 | Spain | Bovine | 352 | 97 | PRJEB1411 |
| UC030 | USA | Human | 97 | 97 | PRJEB1411 |
| VETBG42 | Portugal | Caprine | 97 | 97 | PRJEB1411 |
| 10668 | Denmark | Human | 97 | 97 | PRJEB1411 |
| 55435 | Denmark | Human | 97 | 97 | PRJEB1411 |
| 76325 | Denmark | Human | 97 | 97 | PRJEB1411 |
| 83075 | Denmark | Human | 97 | 97 | PRJEB1411 |
| ED133 | France | Ovine | 133 | 133 | NC_017337 |
| LGA251 | UK | Bovine | 425 | 425 | NC_017349 |
| RF122 | Ireland | Bovine | 151 | 151 | NC_007622 |
| ERR144792 | Denmark | Bovine | 130 | 130 | ERR144792 |
| 11819-97 | Denmark | Human | 80 | 80 | NC_017351 |
| HO50960412 | UK | Human | 22 | 22 | NC_017763 |
| N315 | Japan | Human | 5 | 5 | NC_002745 |
| COL | UK | Human | 8 | 8 | NC_002951 |
| 04-02981 | Germany | Human | 225 | 225 | NC_017340 |
| TCH1516 | USA | Human | 8 | 8 | NC_010079 |
| MW2 | USA | Human | 1 | 1 | NC_003923 |
| M013 | Taiwan | Human | 59 | 59 | NC_016928 |
| JKD6159 | Australia | Human | 93 | 93 | NC_017338 |

| | | | | | |
|---|---|---|---|---|---|
| S0385 | Netherlands | Human | 398 | 398 | NC_017333 |
| JKD6008 | New Zealand | Human | 239 | 239 | NC_017341 |
| MN8 | USA | Human | 30 | 30 | NZ_CM000952 |
| MRSA252 | UK | Human | 36 | 30 | NC_002952 |
| CA-347 | USA | Human | 45 | 45 | NC_021554 |
| MSHR1132 | Australia | Human | 75 | 75 | NC_016941 |

**Table S2. Gene content of predicted recombinant fragments.**

| Recombinant region/ intervening region (IR) | Start | Finish | Gene name | Product |
|---|---|---|---|---|
| 1 | 46 | 522 | fda | Fructose-bisphosphate aldolase class 1 |
| 1 | 715 | 2211 | mqo2 | Probable malate:quinone oxidoreductase 2 |
| 2 | 146 | 1765 | acsA | Acetyl-coenzyme A synthetase |
| 2 | 1938 | 2381 | hmoB | Heme-degrading monooxygenase HmoB |
| 2 | 2661 | 2873 | | hypothetical protein |
| 2 | 3151 | 4860 | betA | Choline dehydrogenase |
| 2 | 5121 | 6611 | gbsA | Betaine aldehyde dehydrogenase |
| 2 | 6863 | 7426 | | hypothetical protein |
| 2 | 7596 | 9218 | opuD | Glycine betaine transporter OpuD |
| 2 | 9736 | 10272 | | anaerobic ribonucleotide reductase-activating protein |
| 2 | 10269 | 12119 | nrdD | Anaerobic ribonucleoside-triphosphate reductase |
| 2 | 12366 | 12611 | citM | Mg(2+)/citrate complex secondary transporter |
| 2 | 12642 | 13736 | citN | Citrate transporter |
| 2 | 14212 | 14817 | sirC | Precorrin-2 dehydrogenase |
| 2 | 14880 | 16760 | cysJ | Sulfite reductase [NADPH] flavoprotein alpha-component |
| 2 | 17155 | 17652 | bsaA | Glutathione peroxidase homolog BsaA |
| 2 | 17662 | 17913 | | hypothetical protein |
| 2 | 19082 | 21082 | bceB_2 | Bacitracin export permease protein BceB |
| 2 | 21079 | 21291 | yxdL_1 | ABC transporter ATP-binding protein YxdL |
| 2 | 21403 | 21834 | yxdL_2 | ABC transporter ATP-binding protein YxdL |
| 2 | 21942 | 22829 | graS | Sensor histidine kinase GraS |

| | | | | |
|---|---|---|---|---|
| 2 | 22840 | 23505 | graR | Glycopeptide resistance-associated protein R |
| 2 | 23531 | 23731 | | hypothetical protein |
| 2 | 23929 | 25353 | phoB | Alkaline phosphatase 3 precursor |
| 2 | 25413 | 25517 | | hypothetical protein |
| 2 | 25636 | 26091 | | transcriptional regulator SlyA |
| 2 | 26336 | 27097 | | S-formylglutathione hydrolase |
| 2 | 27379 | 29259 | clfB | Fibrinogen-binding protein B |
| 2 | 29609 | 30313 | arcR | HTH-type transcriptional regulator ArcR |
| 2 | 30412 | 31353 | arcC2 | Carbamate kinase 2 |
| 2 | 31370 | 32800 | arcD | Arginine/ornithine antiporter |
| 2 | 32891 | 33901 | arcB | Ornithine carbamoyltransferase%2C catabolic |
| 2 | 33934 | 35169 | arcA | Arginine deiminase |
| 2 | 35516 | 35965 | argR | Arginine repressor |
| 2 | 36332 | 37861 | aur | Zinc metalloproteinase aureolysin precursor |
| 2 | 38285 | 38830 | isaB | Immunodominant staphylococcal antigen B precursor |
| 2 | 39069 | 39527 | | hypothetical protein |
| 2 | 39767 | 41641 | licR | Probable licABCH operon regulator |
| 3 | 86 | 481 | manP | EIIBCA-Man |
| 4 | 230 | 754 | yvyI | Putative mannose-6-phosphate isomerase YvyI |
| 4 | 866 | 3847 | | YhgE/Pip C-terminal domain |
| 4 | 4058 | 5917 | | N-acetylmuramoyl-L-alanine amidase domain-containing protein |
| 4 | 6184 | 6744 | | N-carbamoylsarcosine amidase |
| 4 | 6915 | 8810 | | hypothetical protein |
| 4 | 9052 | 10410 | gtf2 | Glycosyltransferase chaperone GtfB |
| 4 | 10403 | 11911 | gtf1 | Glycosyltransferase Gtf1 |
| 4 | 11929 | 14319 | | preprotein translocase subunit SecA |
| 4 | 14309 | 14707 | | hypothetical protein |
| 4 | 14704 | 15267 | asp3 | Orf3 |

| | | | | |
|---|---|---|---|---|
| 4 | 15245 | 16816 | asp2 | Orf2 |
| 4 | 16803 | 18356 | | accessory Sec system protein Asp1 |
| 4 | 18367 | 19578 | | preprotein translocase subunit SecY |
| 4 | 20188 | 21036 | | hypothetical protein |
| 4 | 21160 | 22338 | | hypothetical protein |
| 4 | 22534 | 23340 | | hypothetical protein |
| 4 | 23518 | 24723 | | hypothetical protein |
| 4 | 25798 | 26028 | | hypothetical protein |
| 4 | 27174 | 27866 | | Flavin reductase like domain |
| 4 | 28207 | 28842 | | hypothetical protein |
| 4 | 28991 | 29350 | | hypothetical protein |
| 4 | 29435 | 29698 | | hypothetical protein |
| 4 | 29733 | 30032 | | hypothetical protein |
| 4 | 30022 | 30360 | | lineage-specific thermal regulator protein |
| 4 | 30527 | 30991 | | hypothetical protein |
| 4 | 31153 | 34143 | cna | Collagen adhesin precursor |
| 4 | 34513 | 35931 | yflS | Putative malate transporter YflS |
| 4 | 36330 | 37238 | | putative chloramphenical resistance permease RarD |
| 4 | 37460 | 38035 | | hypothetical protein |
| 4 | 38201 | 39217 | nixA | High-affinity nickel-transport protein NixA |
| 4 | 39460 | 40272 | nhoA | N-hydroxyarylamine O-acetyltransferase |
| 4 | 40229 | 40513 | | hypothetical protein |
| 4 | 40510 | 40650 | | hypothetical protein |
| 4 | 40647 | 41159 | | putative conserved protein |
| 4 | 41449 | 42207 | bceA | Bacitracin export ATP-binding protein BceA |
| 4 | 42197 | 44077 | bceB_1 | Bacitracin export permease protein BceB |
| 4 | 44169 | 44360 | | hypothetical protein |
| 4 | 44633 | 45115 | | Transposase and inactivated derivatives%2C IS30 family |

| | | | | |
|---|---|---|---|---|
| 4 | 45450 | 45947 | | hypothetical protein |
| 4 | 45949 | 46698 | | hypothetical protein |
| 4 | 46717 | 47520 | apr | Subtilisin DY |
| 4 | 48004 | 48204 | cspC | Cold shock protein CspC |
| 4 | 48331 | 48900 | immR | HTH-type transcriptional regulator ImmR |
| 4 | 49160 | 49555 | | hypothetical protein |
| 4 | 49623 | 49976 | | hypothetical protein |
| 4 | 50293 | 51132 | noc | Nucleoid occlusion protein |
| 4 | 51175 | 51894 | rsmG | Ribosomal RNA small subunit methyltransferase G |
| 4 | 51894 | 53771 | mnmG | Glucose-inhibited division protein A |
| 4 | 53838 | 55217 | mnmE | tRNA modification GTPase MnmE |
| 4 | 55360 | 55713 | rnpA | Ribonuclease P protein component |
| 4 | 55834 | 55971 | rpmH | 50S ribosomal protein L34 |
| 6 | 19 | 1053 | dnaA | Chromosomal replication initiator protein DnaA |
| 7 | 477 | 713 | | S4 domain protein YaaA |
| 7 | 710 | 1822 | recF | DNA replication and repair protein RecF |
| 7 | 1832 | 3766 | gyrB | DNA gyrase subunit B |
| 7 | 3803 | 6463 | gyrA | DNA gyrase subunit A |
| 7 | 6551 | 7363 | nnrD | ADP-dependent (S)-NAD(P)H-hydrate dehydratase |
| 7 | 7689 | 9203 | hutH | Histidine ammonia-lyase |
| 7 | 9581 | 10867 | serS | Serine--tRNA ligase |
| 7 | 11520 | 12215 | ygaZ | Inner membrane protein YgaZ |
| 7 | 12212 | 12541 | | putative membrane protein |
| 7 | 12904 | 13872 | metX | Homoserine O-acetyltransferase |
| 7 | 14180 | 15103 | | putative membrane protein |
| 7 | 15118 | 17085 | nrnA | Bifunctional oligoribonuclease and PAP phosphatase NrnA |
| 7 | 17082 | 17528 | rplI | 50S ribosomal protein L9 |
| 7 | 17560 | 18960 | dnaC | Replicative DNA helicase |

| | | | | |
|---|---|---|---|---|
| 7 | 19239 | 20522 | purA | Adenylosuccinate synthetase |
| 7 | 21346 | 22047 | walR | Transcriptional regulatory protein WalR |
| 7 | 22060 | 23886 | walK | Sensor protein kinase WalK |
| 7 | 23879 | 25213 | | YycH protein |
| 7 | 25214 | 26002 | yycI | Two-component system YycFG regulatory protein |
| 7 | 26400 | 27191 | yycJ | Putative metallo-hydrolase YycJ |
| 7 | 27418 | 29736 | yfkN_1 | Trifunctional nucleotide phosphoesterase protein YfkN precursor |
| 7 | 30104 | 30583 | rlmH | Ribosomal RNA large subunit methyltransferase H |
| 7 | 30695 | 31885 | | hypothetical protein |
| 7 | 32441 | 32614 | | putative transposase OrfB |
| 7 | 33134 | 33793 | | hypothetical protein |
| 7 | 33936 | 34367 | | Transposase and inactivated derivatives |
| 7 | 34920 | 35078 | | hypothetical protein |
| 7 | 35456 | 36235 | sely | SElY |
| 7 | 36420 | 36797 | dus_1 | Probable tRNA-dihydrouridine synthase |
| 7 | 37089 | 37844 | | Sulfite exporter TauE/SafE |
| 7 | 37844 | 38104 | csoR | Copper-sensitive operon repressor |
| 7 | 38241 | 39308 | glpE | Thiosulfate sulfurtransferase GlpE |
| 7 | 39339 | 40673 | blh | Beta-lactamase hydrolase-like protein |
| 7 | 40691 | 41884 | fccB | Sulfide dehydrogenase [flavocytochrome c] flavoprotein chain precursor |
| 7 | 42535 | 43209 | dus_2 | Probable tRNA-dihydrouridine synthase |
| 7 | 43518 | 43721 | | hypothetical protein |
| 7 | 43771 | 44067 | | Regulator of competence-specific genes |
| 7 | 44130 | 45350 | | enterobactin exporter EntS |
| 7 | 45343 | 45894 | ywqN | Putative NAD(P)H-dependent FMN-containing oxidoreductase YwqN |
| 7 | 46007 | 46867 | gltR | HTH-type transcriptional regulator GltR |
| 7 | 47469 | 47951 | | hypothetical protein |
| 7 | 48156 | 49142 | plc | 1-phosphatidylinositol phosphodiesterase precursor |

| | | | | |
|---|---|---|---|---|
| 7 | 49363 | 50130 | | Uncharacterized lipoprotein SAV2485 precursor |
| 7 | 50228 | 52465 | btr | Bacillibactin transport regulator |
| 7 | 52616 | 53794 | yxeP | Uncharacterized hydrolase YxeP |
| 7 | 53796 | 55184 | norB_1 | Quinolone resistance protein NorB |
| 7 | 55672 | 57339 | | Na/Pi-cotransporter II-related protein |
| 7 | 57658 | 59433 | | Oleate hydratase |
| 7 | 59607 | 60479 | eamA | Probable amino-acid metabolite efflux pump |
| 7 | 60686 | 61990 | norG | HTH-type transcriptional regulator NorG |
| 7 | 62180 | 62653 | | putative membrane protein |
| 7 | 62915 | 64507 | lctP | L-lactate permease |
| 7 | 64836 | 65282 | spa_1 | Staphylococcal protein A |
| 7 | 65260 | 65991 | spa_2 | Staphylococcal protein A |
| 7 | 66376 | 67128 | sarS | Staphylococcal accessory regulator S |
| 7 | 67496 | 68494 | yfhA | Probable siderophore transport system permease protein YfhA |
| 7 | 68491 | 69486 | yfiZ | Probable siderophore transport system permease protein YfiZ precursor |
| 7 | 69502 | 70494 | yfiY | Probable siderophore-binding lipoprotein YfiY precursor |
| 7 | 70725 | 71705 | sbnA | Probable siderophore biosynthesis protein SbnA |
| 7 | 71702 | 72712 | | alanine dehydrogenase |
| 7 | 72733 | 74487 | iucC_1 | Aerobactin synthase |
| 7 | 74480 | 75736 | tetA | Metal-tetracycline/H(+) antiporter |
| 7 | 75726 | 77462 | iucA | N(2)-citryl-N(6)-acetyl-N(6)-hydroxylysine synthase |
| 7 | 77443 | 79221 | iucC_2 | Aerobactin synthase |
| 7 | 79196 | 79972 | garL | 5-keto-4-deoxy-D-glucarate aldolase |
| 7 | 79972 | 81174 | btrK | L-glutamyl-[BtrI acyl-carrier protein] decarboxylase |
| 7 | 81178 | 81942 | | ParB-like nuclease domain |
| 7 | 82144 | 82566 | | hypothetical protein |
| 7 | 82616 | 83410 | | hypothetical protein |
| 7 | 83620 | 84396 | butA | Diacetyl reductase [(S)-acetoin forming] |

| | | | | |
|---|---|---|---|---|
| 7 | 84719 | 85711 | | UDP-glucose 4-epimerase |
| 7 | 85674 | 86366 | tuaA | Putative undecaprenyl-phosphate N-acetylgalactosaminyl 1-phosphate |
| 7 | 86576 | 87742 | epsD | Putative glycosyltransferase EpsD |
| 7 | 87723 | 88961 | | Lipid A core - O-antigen ligase and related enzymes |
| 7 | 88951 | 90381 | rfbX | Putative O-antigen transporter |
| 7 | 90649 | 91248 | sodM | Superoxide dismutase [Mn/Fe] 2 |
| 7 | 91676 | 92416 | treR | Trehalose operon transcriptional repressor |
| 7 | 92667 | 93374 | deoD | Purine nucleoside phosphorylase DeoD-type |
| 7 | 93375 | 94733 | norB_2 | Quinolone resistance protein NorB |
| 7 | 94814 | 95509 | deoC1 | Deoxyribose-phosphate aldolase 1 |
| 7 | 95570 | 96748 | deoB | Phosphopentomutase |
| 7 | 96878 | 97693 | phnE_1 | Phosphate-import permease protein PhnE |
| 7 | 97690 | 98490 | phnE_2 | Phosphate-import permease protein PhnE |
| 7 | 98492 | 99265 | glnQ | Glutamine transport ATP-binding protein GlnQ |
| 7 | 99479 | 100435 | phnD | Phosphate-import protein PhnD precursor |
| 7 | 100664 | 102208 | | ABC-type uncharacterized transport system |
| 7 | 102259 | 103794 | yfkN_2 | Trifunctional nucleotide phosphoesterase protein YfkN precursor |
| 7 | 103951 | 104718 | | hypothetical protein |
| 7 | 104724 | 105899 | | Helix-turn-helix |
| 7 | 106285 | 108894 | adhE | Aldehyde-alcohol dehydrogenase |
| 7 | 109239 | 109907 | cap8A | Capsular polysaccharide type 8 biosynthesis protein cap8A |
| 7 | 109923 | 110609 | cap8B | Capsular polysaccharide type 8 biosynthesis protein cap8B |
| 7 | 110612 | 111376 | cap8C | Capsular polysaccharide type 8 biosynthesis protein cap8C |
| 7 | 111396 | 113219 | cap8D | Capsular polysaccharide type 8 biosynthesis protein cap8D |
| 7 | 113209 | 114237 | cap8E | Capsular polysaccharide type 8 biosynthesis protein cap8E |
| 7 | 114250 | 115359 | cap8F | Capsular polysaccharide type 8 biosynthesis protein cap8F |
| 7 | 115363 | 116487 | cap8G | Capsular polysaccharide type 8 biosynthesis protein cap8G |
| 7 | 116490 | 117569 | cap8H | Capsular polysaccharide type 8 biosynthesis protein cap8H |

| | | | | |
|---|---|---|---|---|
| 7 | 117562 | 118956 | cap8I | Capsular polysaccharide type 8 biosynthesis protein cap8I |
| 7 | 118953 | 119510 | cap8J | Capsular polysaccharide type 8 biosynthesis protein cap8J |
| 7 | 119519 | 120757 | cap8K | Capsular polysaccharide type 8 biosynthesis protein cap8K |
| 7 | 120791 | 121996 | cap8L | Capsular polysaccharide type 8 biosynthesis protein cap8L |
| 7 | 122007 | 122564 | cap8M | Capsular polysaccharide type 8 biosynthesis protein cap8M |
| 7 | 122564 | 123451 | cap8N | Capsular polysaccharide type 8 biosynthesis protein cap8N |
| 7 | 123505 | 124767 | cap8O | Capsular polysaccharide type 8 biosynthesis protein cap8O |
| 7 | 124844 | 125989 | cap8P | Capsular polysaccharide type 8 biosynthesis protein cap8P |
| 7 | 126054 | 126380 | isdI | Heme-degrading monooxygenase 2 |
| 7 | 126387 | 126770 | ybaN | Inner membrane protein YbaN |
| 7 | 127197 | 128684 | aldA | Putative aldehyde dehydrogenase AldA |
| 7 | 129332 | 130291 | czcD | Cadmium%2C cobalt and zinc/H(+)-K(+) antiporter |
| 7 | 130352 | 130555 | | hypothetical protein |
| 7 | 130734 | 131246 | | hypothetical protein |
| 7 | 131587 | 132327 | cmpC_1 | Bicarbonate transport ATP-binding protein CmpC |
| 7 | 132341 | 133315 | cmpC_2 | Bicarbonate transport ATP-binding protein CmpC |
| 7 | 133312 | 134073 | ssuC | Putative aliphatic sulfonates transport permease protein SsuC |
| 7 | 134083 | 135117 | | hypothetical protein |
| 7 | 135627 | 136865 | | putative transporter |
| 7 | 137323 | 144498 | grsB | Gramicidin S synthase II |
| 7 | 144511 | 145155 | psf-1 | 4'-phosphopantetheinyl transferase psf-1 |
| 7 | 145483 | 145977 | yagU | Inner membrane protein YagU |
| 7 | 146247 | 147017 | argB | Acetylglutamate kinase |
| 7 | 147033 | 148274 | argJ | Arginine biosynthesis bifunctional protein ArgJ |
| 7 | 148286 | 149320 | argC | N-acetyl-gamma-glutamyl-phosphate reductase |
| 7 | 149356 | 150540 | rocD2 | Ornithine aminotransferase 2 |
| 7 | 150793 | 152148 | brnQ_1 | LIV-II |
| 7 | 152426 | 152983 | yecD | Isochorismatase family protein YecD |

| | | | | |
|---|---|---|---|---|
| 7 | 153049 | 154689 | ipdC | Indole-3-pyruvate decarboxylase |
| 7 | 154820 | 154954 | | hypothetical protein |
| 7 | 154961 | 157006 | ptsG_1 | EIICBA-Glc |
| 7 | 157592 | 158647 | | putative conserved protein |
| 7 | 158647 | 159543 | murQ | N-acetylmuramic acid 6-phosphate etherase |
| 7 | 159555 | 161009 | | PTS system EIIBC component SA0186 |
| 7 | 161009 | 161887 | ybbH | Uncharacterized HTH-type transcriptional regulator YbbH |
| 7 | 162082 | 162363 | | hypothetical protein |
| 7 | 162576 | 165275 | hsdR_1 | Type-1 restriction enzyme R protein |
| 7 | 165272 | 165364 | hsdR_2 | Type-1 restriction enzyme R protein |
| 7 | 165569 | 166411 | | hypothetical protein |
| 7 | 166413 | 167336 | | hypothetical protein |
| 7 | 167323 | 168435 | | RES domain |
| 7 | 168700 | 170292 | gsiA | Glutathione import ATP-binding protein GsiA |
| 7 | 170294 | 171727 | gsiC | Glutathione transport system permease protein GsiC |
| 7 | 171733 | 172896 | gsiD | Glutathione transport system permease protein GsiD |
| 7 | 172913 | 174688 | | ABC-type oligopeptide transport system%2C periplasmic component |
| 7 | 174726 | 176732 | ggt | Gamma-glutamyltranspeptidase precursor |
| 7 | 177270 | 177674 | azoR_1 | FMN-dependent NADH-azoreductase |
| 7 | 177675 | 177896 | azoR_2 | FMN-dependent NADH-azoreductase |
| 7 | 178105 | 178683 | | Glycyl-glycine endopeptidase ALE-1 precursor |
| 7 | 179066 | 180163 | ugpC | sn-glycerol-3-phosphate import ATP-binding protein UgpC |
| 7 | 180176 | 181447 | cycB | Cyclodextrin-binding protein precursor |
| 7 | 181450 | 182718 | malF | Maltose transport system permease protein MalF |
| 7 | 182720 | 183559 | malG | Maltose transport system permease protein MalG |
| 7 | 183736 | 184812 | ycjS_1 | Uncharacterized oxidoreductase YcjS |
| 7 | 184837 | 185877 | ycjS_2 | Uncharacterized oxidoreductase YcjS |
| 7 | 185932 | 186900 | | Hydroxypyruvate isomerase |

| | | | | |
|---|---|---|---|---|
| 7 | 187260 | 187754 | | Putative protein-S-isoprenylcysteine methyltransferase |
| 7 | 187987 | 189366 | uhpT | Hexose phosphate transport protein |
| 7 | 189726 | 190484 | | Uncharacterized response regulatory protein SACOL0201 |
| 7 | 190477 | 192033 | | Uncharacterized sensor-like histidine kinase SACOL0202 |
| 7 | 192030 | 192998 | | 2-aminoethylphosphonate ABC transporter substrate-binding protein |
| 7 | 193586 | 195835 | pflB | Formate acetyltransferase |
| 7 | 195858 | 196613 | pflA | Pyruvate formate-lyase-activating enzyme |
| 7 | 196934 | 198697 | | cytoplasmic glycerophosphodiester phosphodiesterase |
| 7 | 198861 | 199205 | scn | Staphylococcal complement inhibitor precursor |
| 7 | 199395 | 201116 | | Staphylocoagulase precursor |
| 7 | 201039 | 201467 | | hypothetical protein |
| 7 | 202077 | 203261 | fadA | 3-ketoacyl-CoA thiolase |
| 7 | 203291 | 205552 | fadN | Probable 3-hydroxyacyl-CoA dehydrogenase |
| 7 | 205737 | 206948 | acdA | Acyl-CoA dehydrogenase |
| 7 | 207060 | 208565 | lcfB | Long-chain-fatty-acid--CoA ligase |
| 7 | 208591 | 210153 | ydiF | Acetate CoA-transferase YdiF |
| 7 | 210522 | 211751 | | hypothetical protein |
| 7 | 212066 | 213541 | gsiB | Glutathione-binding protein GsiB precursor |
| 7 | 213739 | 214095 | | putative conserved protein |
| 7 | 214247 | 214420 | | hypothetical protein |
| 7 | 214446 | 215591 | hmp | Nitric oxide dioxygenase |
| 7 | 216164 | 217117 | ldhA | L-lactate dehydrogenase 1 |
| 7 | 217438 | 218967 | ptsG_2 | EIICBA-Glc |
| 7 | 219329 | 220264 | rihA | Pyrimidine-specific ribonucleoside hydrolase RihA |
| 7 | 220602 | 221630 | mtlR | Mannitol operon transcriptional activator |
| 7 | 221673 | 222698 | ulaC | Ascorbate-specific phosphotransferase enzyme IIA component |
| 7 | 222683 | 223150 | fruA | EIIABC-Fru |
| 7 | 223173 | 223451 | gatB | Galactitol-specific phosphotransferase enzyme IIB component |

| | | | | |
|---|---|---|---|---|
| 7 | 223678 | 224937 | gatC | PTS system galactitol-specific EIIC component |
| 7 | 224955 | 226010 | | D-arabitol-phosphate dehydrogenase |
| 7 | 226012 | 226158 | | hypothetical protein |
| 7 | 226182 | 227225 | gatD | Galactitol-1-phosphate 5-dehydrogenase |
| 7 | 227754 | 228470 | ispD1 | 2-C-methyl-D-erythritol 4-phosphate cytidylyltransferase 1 |
| 7 | 228463 | 229488 | gutB | Sorbitol dehydrogenase |
| 7 | 229510 | 230823 | tagB_1 | Putative CDP-glycerol:glycerophosphate glycerophosphotransferase |
| 7 | 230865 | 231293 | tagB_2 | Putative CDP-glycerol:glycerophosphate glycerophosphotransferase |
| 7 | 231868 | 233037 | tagF | CDP-glycerol:poly(glycerophosphate) glycerophosphotransferase |
| 7 | 233313 | 234029 | ispD2 | 2-C-methyl-D-erythritol 4-phosphate cytidylyltransferase 2 |
| 7 | 234022 | 235047 | idnD | L-idonate 5-dehydrogenase |
| 7 | 235069 | 235980 | | hypothetical protein |
| 7 | 236126 | 236752 | tagB_3 | Putative CDP-glycerol:glycerophosphate glycerophosphotransferase |
| 7 | 236785 | 238506 | epsJ | Uncharacterized glycosyltransferase EpsJ |
| 7 | 238650 | 239324 | scdA | Iron-sulfur cluster repair protein ScdA |
| 7 | 239570 | 241324 | ypdA | Sensor histidine kinase YpdA |
| 7 | 241327 | 242067 | lytR | Sensory transduction protein LytR |
| 7 | 242186 | 242623 | lrgA | Antiholin-like protein LrgA |
| 7 | 242616 | 243317 | lrgB | Antiholin-like protein LrgB |
| 7 | 243658 | 245940 | norB_3 | Nitric oxide reductase subunit B |
| 7 | 246175 | 246879 | yydK | Uncharacterized HTH-type transcriptional regulator YydK |
| 7 | 247028 | 247258 | | PTS system%2C beta-glucoside-specific IIABC component |
| 7 | 247271 | 247756 | ptsG_3 | EIICBA-Glc 1 |
| 7 | 247843 | 249279 | bglA | Aryl-phospho-beta-D-glucosidase BglA |
| 7 | 249343 | 249528 | | hypothetical protein |
| 7 | 249761 | 250522 | rebM | Demethylrebeccamycin-D-glucose O-methyltransferase |
| 7 | 250773 | 251687 | rbsK | Ribokinase |
| 7 | 251715 | 252119 | rbsD | D-ribose pyranase |

| | | | | |
|---|---|---|---|---|
| 7 | 252134 | 253015 | glcU | Probable glucose uptake protein GlcU |
| 7 | 253247 | 254245 | degA | Degradation activator |
| 7 | 254626 | 255018 | | hypothetical protein |
| 7 | 255147 | 256523 | norB_4 | Quinolone resistance protein NorB |
| 7 | 256757 | 257086 | cbh | Choloylglycine hydrolase |
| 7 | 257105 | 257749 | | Penicillin acylase precursor |
| 7 | 258075 | 259022 | lytM | Glycyl-glycine endopeptidase LytM precursor |
| 7 | 259075 | 259734 | ybhF | Uncharacterized ABC transporter ATP-binding protein YbhF |
| 7 | 259748 | 260668 | | ABC-2 family transporter protein |
| 7 | 260665 | 261831 | | ABC-2 family transporter protein |
| 7 | 261899 | 263422 | | hypothetical protein |
| 7 | 263753 | 264646 | ssaA2 | Staphylococcal secretory antigen ssaA2 precursor |
| 7 | 264894 | 265187 | esxA | Virulence factor EsxA |
| 8 | 62 | 520 | | type VII secretion protein EssA |
| 8 | 492 | 734 | | putative small protein |
| 8 | 49 | 225 | | hypothetical protein |
| IR3 | 28 | 4188 | eccCa1 | ESX-1 secretion system protein EccCa1 |
| IR3 | 4218 | 4610 | | hypothetical protein |
| IR3 | 4626 | 4940 | | Virulence factor EsxB |
| IR3 | 4937 | 5614 | | hypothetical protein |
| IR3 | 5614 | 5931 | | hypothetical protein |
| IR3 | 5941 | 7785 | yeeF | Putative ribonuclease YeeF |
| IR3 | 7796 | 8035 | yezG_1 | Probable antitoxin YezG |
| IR3 | 8010 | 8534 | yezG_2 | Probable antitoxin YezG |
| 9 | 93 | 467 | | hypothetical protein |
| 9 | 617 | 1015 | | hypothetical protein |
| 9 | 1265 | 2089 | focA | Formate channel 1 |
| 9 | 2323 | 3633 | brnQ_2 | LIV-II |

| | | | | |
|---|---|---|---|---|
| 9 | 4217 | 5107 | hel | Outer membrane protein P4 |
| 9 | 2 | 982 | | ABC exporter transmembrane subunit DevC protein |
| 10 | 995 | 1672 | lolD | Lipoprotein-releasing system ATP-binding protein LolD |
